# Machine learning framework for predicting susceptibility to obesity

**DOI:** 10.1038/s41598-025-20505-9

**Published:** 2025-10-08

**Authors:** Warda M. Shaban, Hossam El-Din Moustafa, Mervat M. El-Seddek

**Affiliations:** 1 Communication and Electronics Engineering Department, Nile Higher Institute for Engineering and Technology, Mansoura, Egypt; 2 Electronics and Communication Engineering Department, Faculty of Engineering, Mansoura University, Mansoura, Egypt; 3 Communication and Electronics Engineering Department, Faculty of Engineering, Horus University, New Damietta, Egypt

**Keywords:** Obesity, Overweight, Artificial intelligence, Feature selection, Bat algorithm, Quantum mechanism, Computational biology and bioinformatics, Mathematics and computing

## Abstract

Obesity, currently the fifth leading cause of death worldwide, has seen a significant increase in prevalence over the past four decades. Timely identification of obesity risk facilitates proactive measures against associated factors. In this paper, we proposed a new machine learning framework for predicting susceptibility to obesity called ObeRisk. The proposed model consists of three main parts, preprocessing stage (PS), feature stage (FS), and obesity risk prediction (OPR). In PS, the used dataset was preprocessed through several processes; filling null values, feature encoding, removing outliers, and normalization. Then, the preprocessed data passed to FS where the most useful features were selected. In this paper, we introduced a new feature selection methodology called entropy-controlled quantum Bat algorithm (EC-QBA), which incorporated two variations to the traditional Bat algorithm (BA): (i) control BA parameters using Shannon entropy and (ii) update BA positions in local search using quantum mechanisms. Then, these selected features fed into several machine learning (ML) algorithms, including LR, LGBM, XGB, AdaBoost, MLP, KNN, and SVM. The final decision was obtained based on the majority voting. Experiment results demonstrated that the proposed EC-QBA outperformed the most recent feature selection methodology in terms of accuracy, precision, sensitivity, and F-measure. It introduced 96% accuracy, 96% precision, 96.5% sensitivity, and 96.25% F-measure. Additionally, experimental results indicated that the EC-QBA with the proposed OPR model delivered the best performance, surpassing modern strategies for predicting obesity by achieving maximum accuracy.

**Supplementary Information:**

The online version contains supplementary material available at 10.1038/s41598-025-20505-9.

## Introduction

Obesity is recognized as a significant factor in human health, associated with numerous serious diseases and disorders, including cardiovascular disease, type 2 diabetes, hypertension, and certain cancers. It impacts individuals of all ages and socioeconomic statuses, leading to both physical and mental health issues, along with heightened healthcare demands^1^.

The World Health Organization (WHO) designates Body Mass Index (BMI) as the principal metric for categorizing overweight and obesity in adults, whereas the classification criteria for children and adolescents differ based on age and gender^1^. This phenomenon is no longer confined to high-income countries; it is swiftly proliferating in low- and middle-income nations, especially among populations with diminished socioeconomic status^2,3^. Additional details regarding age-specific BMI classifications and WHO criteria have been included in the Supplementary Material.

Despite the severity of this issue, early interventions, a healthy diet, and regular physical activity can largely prevent obesity and its health complications^4,5^. Early detection of risk factors also effectively motivates individuals to adopt a healthy lifestyle. With the increasing availability of health data, Artificial Intelligence (AI) and Machine Learning (ML) technologies are becoming promising tools to support early detection and appropriate treatment decisions^6^.

Machine learning (ML) is a subfield of Artificial Intelligence (AI) that focuses on creating algorithms and statistical models that let computers learn and develop better at tasks over time^7,8^. These algorithms and models use data to learn on their own and develop better at making predictions or decisions without needing to be told what to do by programmers^9,10^. Now, AI and ML have emerged as transformative forces in the healthcare industry and the broader domain of medical computing.

**Fig. 1:**
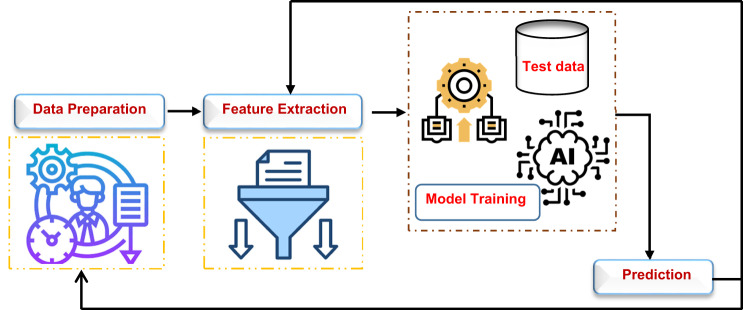
The mechanism of ML.

These technologies are advancing several critical research domains, including disease diagnosis, prognosis, detection, and prediction, as well as analyzing hospital readmission rates, forecasting mortality rates, and monitoring medical wearable devices^11,12^. The emergence of advanced ML techniques and the availability of lifestyle data present an opportunity to develop effective obesity prediction models^13^. Utilizing sophisticated algorithms in ML and AI enables the construction of models that can identify individuals at risk of obesity. These models can examine extensive datasets, discern patterns, and generate precise predictions. Figure 1 shows how ML works.

Elevated data dimensionality is a common issue encountered in ML. Consequently, ML necessitates substantial memory, often containing extraneous or superfluous data, which can lead to overfitting issues^14^. Consequently, feature selection is implemented to address this issue. Feature selection involves eliminating less informative features and identifying the most informative ones^15,16^. Learning effective classifiers for numerous classification problems is exceedingly challenging without the prior elimination of redundant features. Eliminating extraneous features can diminish the complexity of learning algorithms. Various feature selection methodologies exist for identifying the most informative features^17^.

Our study focuses on introducing a new system based on ML to classify weight levels accurately based on information about people, including their gender, age, height, weight, family history of being overweight, eating habits, physical activity, mode of transportation, and the level of obesity that goes with these factors. The key contributions of this paper are summarized as follows:

- Introducing ObeRisk: A Machine Learning Framework for Predicting Obesity Susceptibility for accurately classifying obesity risk levels based on personal, behavioral, and lifestyle data.
- ObeRisk consists of three main stages: Pre-processing Stage (PS), Feature Stage (FS), and Obesity Risk Prediction (ORP).
- A new feature selection methodology was proposed called Entropy-Controlled Quantum Bat Algorithm (EC-QBA), which:
- Integrates Shannon entropy to dynamically control BA parameters.
- Incorporates quantum-inspired mechanisms to improve solution diversity and avoid local optima.
- The ObeRisk framework creates an ensemble prediction methodology that integrates various ML models such as LR, LGBM, XGB, AdaBoost, MLP, KNN, and SVM, and employs majority voting to enhance classification efficacy.
- The experimental evaluation indicated that the ObeRisk framework outperformed both baseline and state-of-the-art methods regarding accuracy, precision, sensitivity, and F-measure, achieving an average accuracy of 97.13%±0.4, precision of 95.7%±0.5, sensitivity of 95.4%±0.4, and F-measure of 95.6%±0.4.

### Paper organization

The organization of the paper is as follows: The problem definition is located in Sect. 2. Section 3 discusses ethical implications of AI. Section 4 provides a summary of prior research. Our framework is presented in Sect. 5. The results are presented in Sect. 6. Conclusions and future work will be discussed in Sect. 7.

## Problem definition

Obesity is one of the most prevalent chronic health problems of modern times, linked to increased rates of certain cancers, heart disease, and type 2 diabetes. Early identification and accurate classification of obesity remain a significant challenge for healthcare systems, especially as the factors influencing obesity, which include genetic, behavioral, nutritional, and environmental factors, increase in complexity, despite advances in diagnostic and treatment methods.

In 2022, obesity affected one in eight individuals worldwide, according to the WHO^1^. The prevalence of obesity in adults has doubled since 1990, while it has quadrupled in adolescents. In that year, 2.5 billion adults aged 18 and older were classified as overweight, with 890 million of them being obese. Additionally, 43% of adults aged 18 and older were classified as overweight, while 16% were considered obese. 35 million children under the age of five were reported to be overweight by 2024. By 2022, over 390 million children and adolescents aged 5–19 were classified as overweight, with 160 million of them being obese^1,3^.

In this context, AI is increasingly important in providing innovative ideas that enable accurate classification of obesity and early prediction of obesity risk. These models enable the development of an intelligent system that helps:

- Early identification of obesity risk.
- Classification of obesity levels: mild, moderate, and severe.
- Proposing early and personalized preventive measures for each patient.

**Fig. 2:**
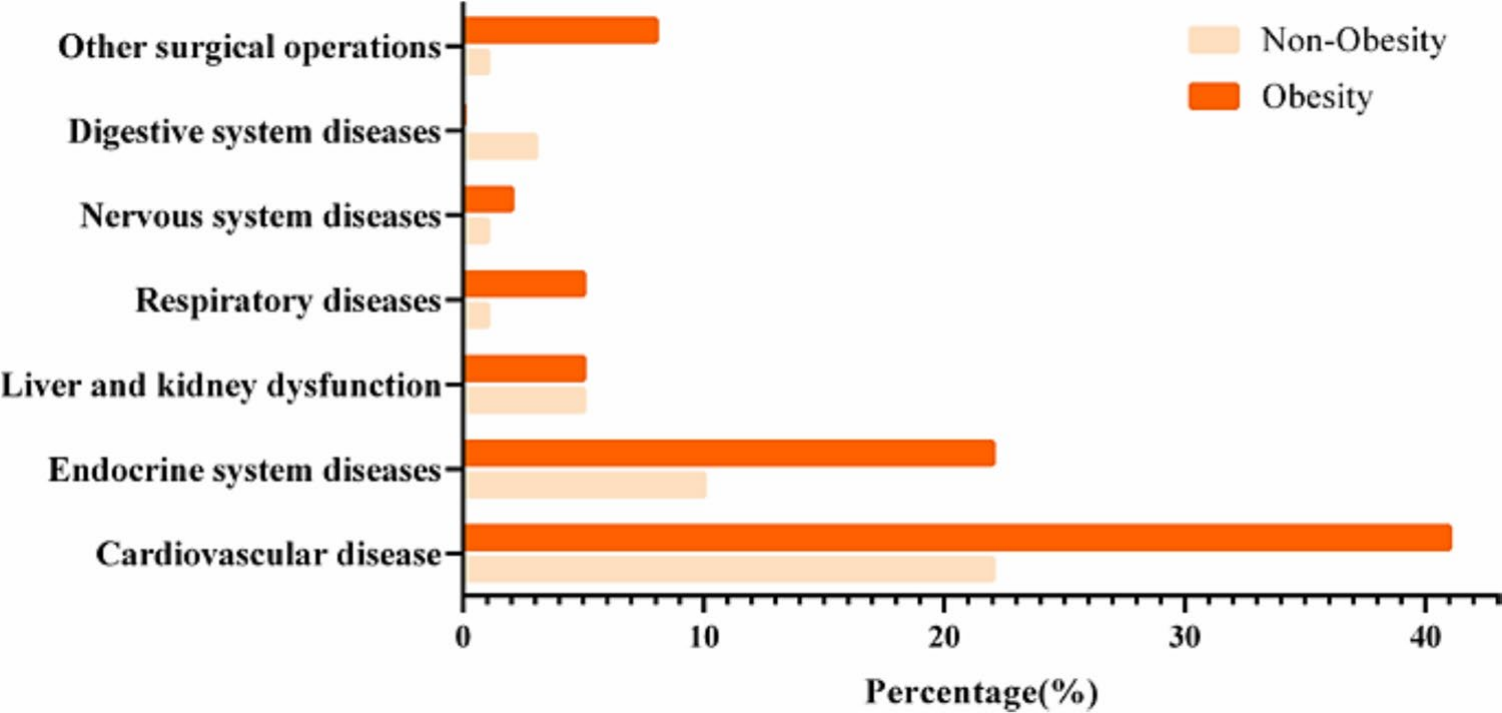
Comparison of fundamental diseases between obese and non-obese patients^18^.

Given the continuous increase in obesity rates worldwide, it is essential to create an intelligent model capable of predicting and classifying obesity risk based on multidimensional health data. With the aim of supporting healthcare providers in making accurate and rapid decisions, thus improving the outcomes of preventive and therapeutic interventions, this study aims to develop a model based on AI techniques to analyze clinical and behavioral data. Figure 2 illustrates the percentage distribution of fundamental diseases in obese and non-obesity patients subjected to statistical analysis.

Consequently, in this paper, a new system is introduced called ObeRisk to predict obesity risk based on AI. As a predictive model, we anticipate that ObeRisk serves as an effective obesity prediction model due to the following reasons: (i) ObeRisk is robust, straightforward, adaptable, rapid, and suitable for real-world applications; (ii) ObeRisk can address challenges associated with imprecise and incomplete data, thereby enabling accurate predictions even with limited training data; (iii) ObeRisk exhibits reduced sensitivity to missing data and demonstrates resilience to noisy data, which mitigates the risk of overfitting; and (iv) when new data or rules are incorporated into the system, retraining is unnecessary; only the addition of new rules is required, alongside a conflict examination for rules. The experimental results demonstrate that the implementation of ObeRisk addresses this issue and outperforms the other recently used methods.

## Ethical implications of AI-driven obesity risk labeling

AI and ML technologies have been effectively utilized in diverse healthcare domains and possess significant potential for predicting and managing obesity through the analysis of intricate health data, identification of risk factors, and assistance to physicians in delivering comprehensive care for obese patients. Through the utilization of AI and ML, patients obtain customized meal plans and an enhanced health monitoring system. Supplementary AI and ML techniques employed in obesity management encompass predictive modeling for evaluating obesity risk.

However, labeling individuals as at risk for obesity can lead to social stigma and discrimination, which can cause psychological harm, including low self-esteem, anxiety, and social isolation. This stigma can discourage individuals from seeking medical care or adopting healthy behaviors, undermining the benefits of this technology. To address these challenges, it is essential to ensure transparency in the prediction process and emphasize that risk classification is probabilistic rather than deterministic, aiming to improve health outcomes rather than simply identify risks. Healthcare providers must be educated to communicate risk information empathetically and involve patients in the decision-making process. Moreover, a regular examination of biases in AI models and the participation of varied stakeholders in their creation can help mitigate undesirable effects.

## Related work

This subsection introduces previous efforts related to AI-based obesity risk prediction. Indeed, the issue of predicting obesity risk has received significant attention recently^11^. As mentioned in^19^, the authors use AI to predict the risk of obesity and make meal plans to help adults lose weight. This study uses a number of ML algorithms, such as Gradient Boosting (GB), Bagging Meta-Estimator (BME), XGBoost (XGB), Random Forest (RF), Support Vector Machine (SVM), and K Nearest Neighbours (KNN), to come up with a complete way to predict the risk of obesity. A dataset is obtained from the UCI ML repository, encompassing features related to the physical characteristics and dietary habits of individuals for the purpose of training the proposed model. According to the obtained results, XGB achieves the highest accuracy.

To calculate obesity rates from lifestyle variables, a Computational Intelligence Model (CIM) was built using supervised and unsupervised data mining techniques, as shown in^20^, such as the Light Gradient Boosting Machine (LightGBM) classifier, RF, Decision Tree (DT), Extremely Randomized Trees (ET), and Logistic Regression (LR). A total of 2,111 participants, ranging in age from fourteen years old to sixty-one, from the countries of Colombia, Mexico, and Peru, were surveyed for this study. Excess calorie consumption, insufficient physical activity, eating disorders, genetic predispositions, and socioeconomic status are some of the primary causes of obesity that are examined in the study. The results show that compared to previous studies conducted under similar circumstances, the LightGBM classification model achieves the best weighted AUC value (0.9990).

In order to better understand the indirect relationships between obesity-related comorbidities and non-communicable diseases, the authors of^21^ lay out the steps necessary to build a thorough Clinical Decision Support System (CDSS) that can predict risk factors associated with these conditions. The graph-based user interface was used to investigate the association of co-occurring disorders with different non-communicable diseases, while ML predictive models are used to analyze the direct correlation between obesity, diabetes, cardiovascular disease, and heart disease. Finally, Explainable Artificial Intelligence (XAI) for interpretation of both local and global models. For the purpose of risk assessment, we offer a variety of ML models and compare them using key performance indicators.

In addition, the study in^22^ seeks to identify risk factors for obesity using ML classifiers and determine the most accurate obesity prediction algorithm. This study uses data from the KNHANES annual survey to assess obesity risk based on blood test results and blood pressure measurements. The dataset has 21,100 participants: 10,000 men and 11,100 women. The obesity prediction was evaluated using six ML algorithms. The results show that obesity risk factors vary by age and gender across ML algorithms. The highest accuracy and AUC for both genders is over 70% in the 19–39 age group, while the 60–79 age group is around 65%. The proposed algorithm had an AUC above 70% in the 40–59 age demographic but below 70% for women. When there are more than six features, the accuracy ratio is similar across classifiers and age groups, but it decreases in the female 19–39 age group.

As illustrated in^23^, it is imperative to promptly identify and mitigate obesity-related health complications. It was suggested that a predictive system be developed to aid individuals in making informed health decisions by providing personalized forecasts for adolescent obesity. The DeepHealthNet system, which is a deep learning framework, effectively trains the model using data augmentation techniques, despite the limited daily health data available. This results in an improved prediction accuracy (acc: 0.8842). Discrepancies in the prediction of obesity rates between boys (accuracy: 0.9320) and girls (accuracy: 0.9163) were identified by the study, which facilitated the identification of disparities and the optimal timing for feedback delivery. The proposed deep learning framework outperformed the alternative general models significantly (*p* < 0.001), as evidenced by statistical analysis. The proposed system is capable of effectively combating obesity in children and adolescents.

To improve individualized and comprehensive obesity healthcare, a visualization-based system for predicting obesity risk was proposed, as described in^24^. The system used a dataset of 1,678 health check-up records that included individual lifestyle variables, body composition, hematological profiles, and biochemical analyses. Ten ML models, including RF and XGBoost, were developed to classify individuals as non-obese (BMI < 25), class 1 obese (25 ≤ BMI < 30), or class 2obese (30 ≤ BMI). The obtained results prove that XGBoost was the most effective model when tested on a test set. Users were able to receive immediate feedback on their obesity risk levels, and the system prioritized interventions based on these results, demonstrating high predictive accuracy and interpretability.

In order to predict the likelihood of obesity using a lifestyle dataset, authors in^25^ used an ensemble learning approach that combined boosting, bagging, and voting techniques. For boosting, XGBoost, Gradient Boosting, and CatBoost models were used. For bagging, Bagged DT, RF, and Extra Tree models was used; and for voting, LR, DT, and SVM models were used. To enhance the data quality assessment, various preprocessing methods were applied. For better prediction results, hyperparameter tuning was utilized along with feature selection and ranking. A wide range of metrics were used to thoroughly analyze and compare the models that have been evaluated.XGBoost outperformed all other models with impressive results: 98.10% accuracy, 97.50% precision and recall, 96.50% F1-score, and 100% AUC-ROC. In addition, the recursive feature elimination method identified and ranked age, height, weight, and body mass index as the most important indicators of the likelihood of obesity.

To precisely assess obesity levels and to elucidate the elements impacting the forecasts, a thorough ML framework was suggested in^26^ that uses Explainable AI (XAI) concepts.In order to incorporate base estimators such as the LightGBM, LR, and RF classifiers—as well as the Stochastic Gradient Descent (SGD) classifier—into the proposed model, an ensemble approach known as a stacking algorithm is utilized. Local Interpretable Model-agnostic Explanations (LIME), a well-established XAI technique, was used to improve the model’s interpretability and reliability. A maximum accuracy of 98.82% was achieved by the suggested framework, according to the results. By incorporating LIME, the reliability of the models was enhanced, and valuable insights into the factors that contribute to the risk of obesity were gained.By bringing model complexity in line with human understanding, the method enhances tailored interventions.

### According to the above literature the research gap can be summarized as follows

- Most models use data that is limited or doesn’t change, and they don’t take into account changes in a person’s health and lifestyle over time or in real time.
- People often don’t think about the psychological, environmental, and social factors that lead to obesity.
- A lot of models don’t make sense, which makes it hard for users and healthcare workers to trust and understand them.
- There isn’t much emphasis on giving personalized health advice or practical steps based on the prediction results.
- Most studies are done on specific areas or groups, so models might not work well for people from other cultures or areas.
- There are differences in how well predictions work for men and women, but they aren’t fully explained.

## The proposed oberisk framework

**Fig. 3:**
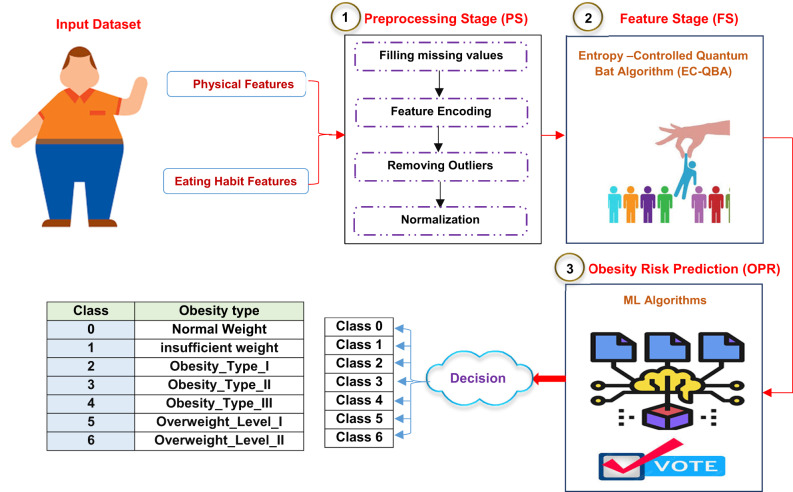
The proposed ObeRisk.

Determining the fundamental causes of obesity risk in its initial phase has become difficult for medical professionals^27,28^. Therefore, in this paper, a new automatic system based on AI is proposed, which is called ObeRisk: Machine Learning Framework for Predicting Obesity Susceptibility. Figure 3 shows the ObeRisk framework. According to Fig. 3, the proposed ObeRisk is composed of several stages: (i) Preprocessing Stage (PS), (ii) Feature Stage (FS), and (iii) Obesity Risk Prediction (ORP). In the next subsections, the detail of each stage will be explained.

### Preprocessing stage (PS)

**Fig. 4:**
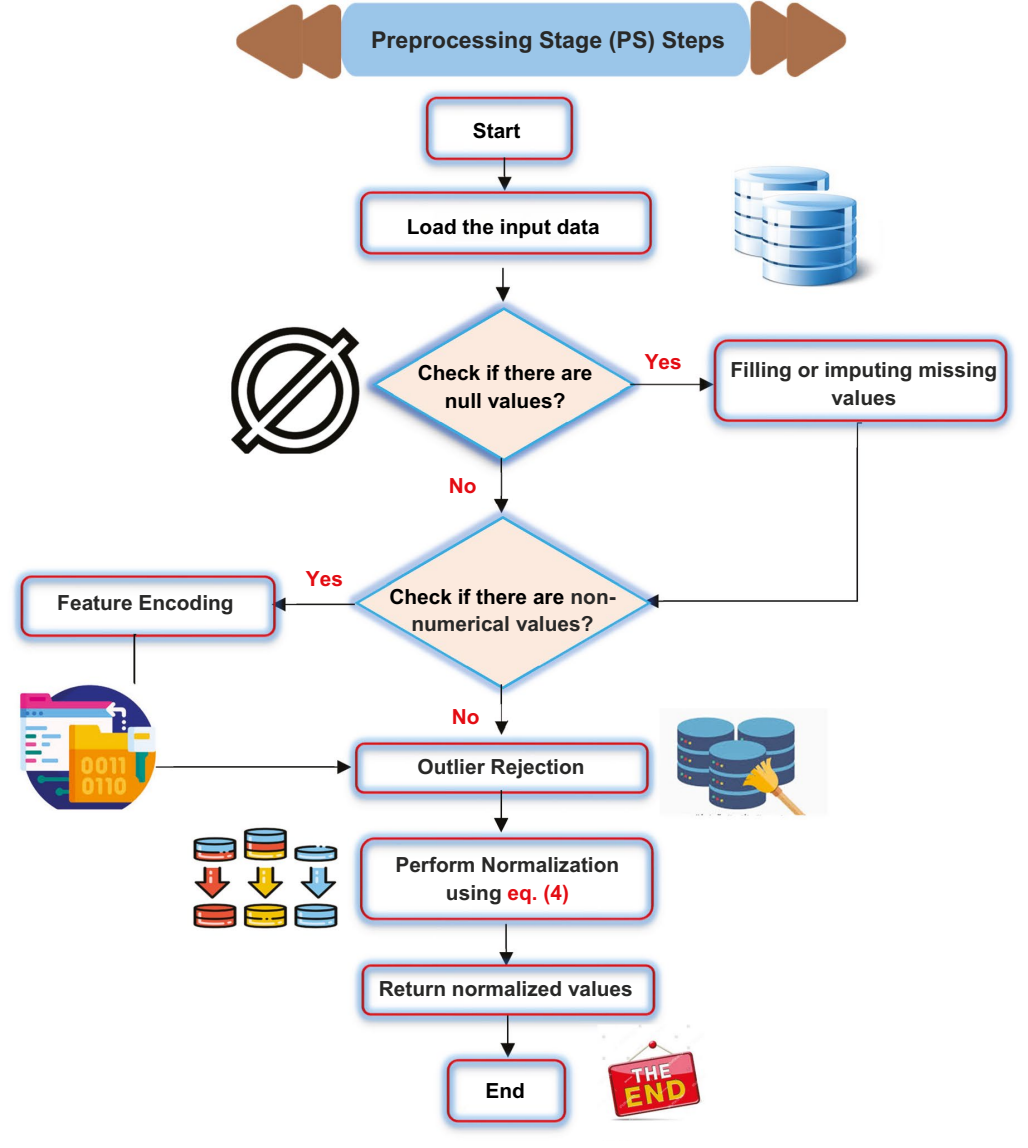
The flowchart of the PS process.

To establish a robust and dependable predictive framework utilizing ML techniques, data preprocessing is of paramount significance and can be performed through Preprocessing Stage (PS)^29^. This serves as a crucial prerequisite that must be performed meticulously prior to progressing to the model construction phase. PS encompasses a sequence of procedures, including filling missing values, feature encoding, removing outliers, and normalization, as shown in (Fig. 4).

In the first step, missing values in the data set used must be dealt with either by removing or filling them in. First, missing values in the data set must be removed or filled in. The following strategy is used to handle the missing values:·.

- Continuous features were filled using mean values of the features of the same class.
- Categorical features were filled out using mode.·.
- Additionally, records with more than 25% of missing values are removed.

Then, feature encoding is a technique used to convert categorical variables into numerical representations. This method is particularly advantageous for algorithms requiring numerical inputs, as most ML models exclusively process numerical data^30^. In this paper, some features are converted variables into numerical using feature encoding, as presented in (Table 1).

**Table 1:** Feature encoding process for some features of the used dataset.

Features	Description	Feature encoding
Gender	Gender of the patients	[0,1]; where 0 for female, and 1 for male
family_history_with_overweight	–	[0,1]; where 0 for No and 1 for Yes
FCVC	Frequency of consumption of vegetables	[0,1]; where 0 for No and 1 for Yes
CAEC	Consumption of food between meals	[0,1,2,3] where 0 for no, 1 for sometimes, 2 for frequently and 3 for always.
Smoke	–	[0,1] ; where 0 for No, and 1 for Yes
SCC	Calories consumption monitoring	[0,1] ; where 0 for No, and 1 for Yes
CALC	Consumption of alcohol	[0,1,2,3] where 0 for no, 1 for sometimes, 2 for frequently and 3 for always.
MTRANS	Transportation used	[0,1,2,3,4] where 0 for walking, 1 for public transport, 2 for bike, 3 for motorbike, and 4 for automobile.
Obesity	Determination of the participant’s obesity status	[0,1,2,3,4,5,6] where 0 for insufficient weight, 1 for normal weight, 2 for obesity type I, 3 for obesity type II, 4 for obesity type III, 5 for overweight level I, and 6 for overweight level II.

The anomalous items are subsequently detected and removed. Removing outliers, especially in predictive modeling, is essential during the preliminary DPS. Outliers are data points that markedly diverge from the other values in a dataset. Proper handling of outliers can significantly impact the effectiveness of prediction models. The Interquartile Range (IQR) method is utilized to identify and replace outliers in the dataset. IQR is the measurement of dispersion between the 25% and 75% of a given data set’s values. These values are (i.e., 25%, 75%) Q1 and Q3 respectively. The following strategy is used to detect and remove outliers^31^:

- Sort the dataset values from the smallest to the largest.
- Calculate IQR using the following equation^31^:

1 $$\:IQR=Q3-Q1$$

- Calculate the lower and upper bound using the following equations:

2 $$\:lb=Q1-1.5*IQR$$

3 $$\:ub=Q3+1.5*IQR$$

Finally, $\:anything<\:lb\:or>ub$ is considered as an outlier item.

The normalization process is an essential component of DPS. The primary objective of normalization is to mitigate the adverse effects of individual data samples by constraining preprocessed data to a defined range^32^. The process of normalization makes gradient descent faster and more accurate. To ensure that the data falls within certain ranges, min-max normalization is often used in this type of study. This is accomplished by performing a linear transformation on the initial data. For the purpose of computing normalized data, the following equation can be used.

4 $$\:{A}_{norm}=\frac{a-{a}_{min}}{\:{a}_{max}-{a}_{min}}$$

Where $\:{A}_{norm}$ is the normalized value, $\:a$ is the initial value. Additionally, the low value is denoted as $\:{a}_{min}$ and the high value is $\:{a}_{max}$.

### Feature stage (FS)

The next stage of our framework is Feature Stage (FS) where the most critical features are identified and prepared to be more suitable for the prediction stage. In FS, the best and most useful features are selected, and the least effective ones are eliminated^33^. The existence of superfluous attributes in the input dataset is a primary contributor to the overfitting issue, particularly in the field of medical diagnosis^34^. It is essential to remove input features that minimally impact the output, as they can diminish the accuracy of the diagnostic model. The feature selection process must be conducted prior to initiating the training of the diagnostic model to enhance its performance, resulting in a more efficient and cost-effective model^35,36^. First, patient characteristics must be extracted from the input dataset, followed by the execution of the feature selection process to identify the most informative attributes.

This paper presents the Entropy Controlled-Quantum Bat Algorithm (EC-QBA), a novel approach to feature selection. The proposed EC-QBA is based on Shannon entropy and quantum mechanism, where each bat parameter, such as frequency, loudness, and pulse rate, will be controlled using normalized entropy. The quantum mechanism was utilized to update the solution during the local search process. Actually, quantum behavior keeps things from converging too soon and lets you explore the feature space more fully, which is excellent for high-dimensional data. Actually, the proposed EC-QBA is based on the Bat Algorithm (BA).

The bio-inspired Bat Algorithm (BA), developed by Xin-She Yang in 2010, is highly efficient. It directly mimics micro-bat echolocation^37,38^. BA is structured around three key characteristics of micro-bats: echolocation behavior, signal frequency $\:f$ with variable wavelength λ, and sound intensity for prey localization^37–39^. For this methodology, Xin-She Yang established the following approximate principles:

(i) Bats use echolocation to measure distance and assess prey proximity.
(ii) To locate prey, bats navigate at a stochastic velocity $\:{V}_{i}$ towards a location $\:{p}_{i}$, producing sounds at a constant frequency $\:{f}_{min}$, with variable wavelength and amplitude $\:{A}_{0}$.
(iii) Based on their target’s proximity, bats can automatically adjust their pulse wavelength and rate.
(iv) The loudness can decrease from $\:{A}_{O}$to $\:{A}_{min}$though it can vary.

In an m-dimensional search space, bats are defined by velocity, position, and frequency. Therefore, the following equations determine the bat’s updated velocity, position, and frequency for the next iteration^39^:

5 $$\:{Vx}_{i}\left(t+1\right)={Vx}_{i}\left(t\right)+\left({x}_{i}\left(t\right)-{x}_{g}\left(t\right)\right){f}_{i}$$

6 $$\:{x}_{i}\left(t+1\right)={x}_{i}\left(t\right)+{Vx}_{i}\left(t+1\right)$$

Where t represents the present cycle and $\:{Vx}_{i}\left(t+1\right)$ denotes the velocity of the $\:{i}^{th}$ bat at the next cycle. $\:{Vx}_{i}\left(t\right)$ signifies the velocity of the $\:{i}^{th}$ bat at the current iteration, whereas $\:{x}_{i}\left(t\right)$ indicates the location of the $\:{i}^{th}$ bat at the present iteration. $\:{x}_{g}\left(t\right)$ represents the present optimal solution within the bat population, whereas $\:{x}_{i}\left(t+1\right)$ represent the location of the $\:{i}^{th}$ bat in the next cycle (t + 1). $\:{f}_{i}$ is the frequency of the $\:{i}^{th}$ bat, which is updated at each cycle as specified in (7)^40^:

7 $$\:{f}_{i}={f}_{min}+\left({f}_{max}-{f}_{min}\right)\beta\:$$

where β is a random variable with a uniform distribution and a value between 0 and 1. After picking a solution from the existing best ones, the algorithm’s local search component uses a random walk to generate a new solution locally for every bat. To accomplish this, we employ Eq. (8)^38^:

8 $$x_{i} \left( {t + 1} \right) = x_{i} \left( t \right) + \varepsilon \bar{A} _{t} ~$$

Where ε be a random variable that falls within the interval [-1,1]. The vector $\bar{A} _{t}$ represents the mean value of $\:A$ for all bats in the present cycle, which is used for exploration instead of exploitation. Bat range and spatial parameters are defined by the BA’s frequency value. When bats get closer to their food source, they slow their pulse emission rate $\:\left(r\right)$ and reduce their intensity $\:\left(A\right)$. The emission pulse rate $\:r\:$and loudness $A$ can be determined using Eqs. (9) and (10)^38^:

9 $$\:{\:A}_{i}\left(t+1\right)=\alpha\:{A}_{i}\left(t\right)$$

10 $$\:{r}_{i}\left(t+1\right)={r}_{i}\left(0\right)\left(1-{e}^{-\gamma\:t}\right)\:$$

Where $\:{\:A}_{i}\left(t+1\right)$denotes the loudness of the $\:{i}^{th}$ bat at the subsequent cycle and $\:{A}_{i}\left(t\right)$ represents the loudness of the $\:{i}^{th}$ bat at the present cycle. Furthermore, $\:{r}_{i}\left(t+1\right)$ denotes the emission pulse rate at the subsequent iteration, while $\:{r}_{i}\left(0\right)$ represents the initial emission pulse rate. $\:\alpha\:$ and $\:\gamma\:$ remain constant.

**Fig. 5:**
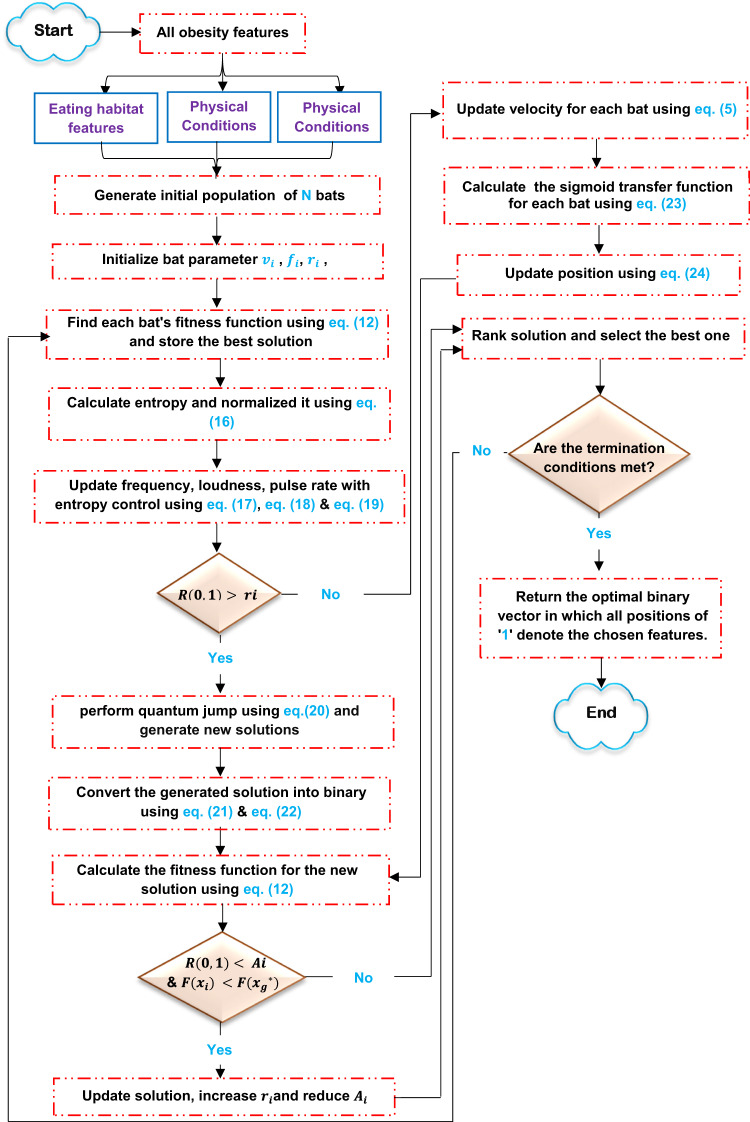
The sequential steps of EC-QBA.

Although the main advantages of behavioral analysis are simplicity, low workload, few parameters, and robustness, it suffers from slow convergence speed and low optimization accuracy. Therefore, this paper presents a new version of behavioral analysis that combines Shannon entropy and quantum mechanism with traditional behavioral analysis. The main advantages of combining Shannon entropy and quantum mechanism with traditional behavioral analysis are improved exploration and exploitation, accelerated convergence, increased ensemble diversity, improved solution accuracy, and scalability and robustness. Figure 5 illustrates the sequential steps of the proposed EC-QBA.

Figure 5 shows that after the PS, the proposed EC-QBA initializes an initial set of $\:N$ bats using the following equation:

11 $$\:{b}_{i}={b}_{min}+\lambda\:\left({b}_{max}-{b}_{min}\right)$$

Where $\:{b}_{i}$ signifies the starting population, where $\:i\:=\:1,\:2,\:\dots\:,\:N$ indicates the population size. $\:{b}_{min}$ denotes the minimum parameter value, while $\:{b}_{max}$ represents the maximum parameter value. Furthermore, initialize the variables $\:{v}_{i}$, $\:{f}_{i}$, and $\:{r}_{i}$.

Next, calculate the fitness value of each bat (i.e., solution) using fitness function using the following equation:

12 $$\:Fitness\:\left({x}_{i}\right)=w*Error\:\left({x}_{i}\right)+(1-w)*\frac{\left|{x}_{i}\right|}{d\:}$$

Where $\:w$ denotes a trade-off parameter; $\:w\:\in\:\left[\text{0,1}\right]$, $\:\left|{x}_{i}\right|$ represents the count of selected features, and $\:d$ signifies the total number of features. $\:Error\:\left({x}_{i}\right)$ denotes the classification error of the selected features, where $\:Error\:\left({x}_{i}\right)=1-Accuracy\:\left({x}_{i}\right)$. $\:Accuracy\:\left({x}_{i}\right)$ can be computed using base classifiers such as KNN, NB, and SVM. This paper employs KNN to assess each solution utilizing Euclidean Distance (ED), which can be computed using the subsequent equation:

13 $$\:ED\left(g,z\right)=\sqrt{\sum\:_{i=1}^{m}\left({z}_{i}-{g}_{i}\right)}\:\:$$

Where $\:{z}_{i}$ and $\:{g}_{i}$ reflect specific attributes of a specific record within the population. $\:i$ is a variable such that $\:i=\text{1,2},...,m$. The proposed method seeks to identify the individual with the highest $\:Fit\left({x}_{i}\right)$. Then, convert each bat fitness into selection probability as illustrated in Eq. (14).

14 $$\:{p}_{i}\left(t\right)=\frac{{e}^{-Fitness\:\left({x}_{i}\right)}}{\sum\:_{i=1}^{N}{e}^{-Fitness\:\left({x}_{i}\right)}}$$

Where $\:{p}_{i}\left(t\right)$ denotes the probability of selecting $\:{i}^{th}$ bat solution based on its fitness at iteration $\:t$. Then, calculate the Shannon entropy using the following equation^39^.

15 $$\:H\left(t\right)=-\sum\:_{i=1}^{N}{p}_{i}\left(t\right).{{log}}_{2}{p}_{i}\left(t\right)\:$$

Where $\:{{log}}_{2}{p}_{i}\left(t\right)$ represents the information content in $\:{i}^{th}$ bat solution. Actually,$\:\:H\left(t\right)$ measures how the bat population diverse at iteration $\:t$. Next, calculate the normalized value $\:{H}_{norm}\left(t\right)$ using Eq. (16).

16 $$\:{H}_{norm}\left(t\right)=\frac{H\left(t\right)}{{log}_{2}\left(N\right)}\:$$

**Fig. 6:**
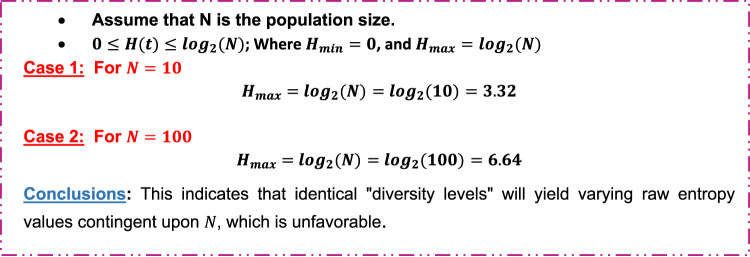
An illustrative example of the importance of using normalized entropy.

Where $\:N$ is the population size. Actually, we use normalized value to make entropy not depend on the size of the population $\:N$. Figure 6 shows an illustrative example of why normalized entropy used.

After calculating normalized entropy, we integrate it to update the frequency, loudness, and pulse rate during each iteration. Entropy tells us if bats are coming together or going apart. The updated parameters can be determined using the following equations:

17 $$\:{f}_{i}={f}_{min}+{H}_{norm}\left({f}_{max}-{f}_{min}\right)$$

18 $$\:{A}_{i}\left(t+1\right)={A}_{i}\left(t\right)\left(1-{H}_{norm}\right)$$

19 $$\:{r}_{i}\left(t+1\right)={r}_{i}\left(t\right)\left(1-{e}^{-\varUpsilon\:{H}_{norm}t}\right)$$

Where $\:{H}_{norm}\left(t\right)$ is the normalized entropy, $\:{H}_{norm}\left(t\right)\in\:\left[\text{0,1}\right]$; where $\:{H}_{norm}\left(t\right)=0$ means all bat are similar (minimum diversity) and $\:{H}_{norm}\left(t\right)=1$ means all bat are equally different (maximum diversity). In fact, using normalized entropy makes the adaptive frequency, loudness, and pulse rate work in a stable way. Additionally using normalized entropy $\:{H}_{norm}\left(t\right)$ has several advantages such as:

(i) Consistency: the entropy measure doesn’t depend on the size of the population.
(ii) Control: makes sure that things like frequency and volume are measured correctly.
(iii) Stability: makes sure that control behavior is smooth across iterations.
(iv) Adaptability: helps keep exploration and exploitation in check.

After that, inspired by quantum mechanism, quantum updating moves the bat closer to the best solution in the world by making a “quantum tunneling”-style jump that is based on chance. Actually, updating position using quantum mechanism is located when local search.

is occurred using the following equation:

20 $$\:{x}_{i}\left(t+1\right)={x}_{g}\left(t\right)+ \theta \left({x}_{i}\left(t\right)-{x}_{g}\left(t\right)\right)ln\left(\frac{1}{u}\right)$$

Where $\:{x}_{i}\left(t+1\right)$ donates the new position of $\:{i}^{th}$ bat at the next cycle$\:\:(t+1)$. $\:{x}_{g}\left(t\right)$ represents the global position and $\:{x}_{i}\left(t\right)$ represents the position of $\:{i}^{th}$ bat at cycle $\:t$. *θ* represents quantum scaling factor that sets the distance the racket jumps; smaller numbers mean smaller steps. $\:u$ represents uniform random number; $\:u\in\:\left[\text{0,1}\right]$. The $\:ln\left(\frac{1}{u}\right)$ step is usually small, but it can also be big, which lets you take advantage of and get away from local optimums. Then, convert the updated position into binary as we deal with a discrete problem (i.e., feature selection) using sigmoid transfer function as illustrated in the following equations^17^.

21 $$\:{s}_{i}^{j}=\frac{1}{1+{e}^{-{x}_{i}}}\:$$

22 $$\:{x}_{i}^{j}\left(t+1\right)=\left\{\begin{array}{c}0\:\:\:\:\:\:\:\:\:\:\:\:\:\:\:if\:\:\:rand\left(\text{0,1}\right)\ge\:{s}_{i}^{j}\\\:\:\\\:\:1\:\:\:\:\:\:\:\:\:\:\:\:\:\:\:\:\:\:\:\:\:otherwise\:\:\:\:\:\:\:\:\:\:\:\:\:\:\:\:\:\:\:\:\:\:\:\:\end{array}\right.$$

Where $\:{x}_{i}^{j}\left(t+1\right)$ represents the $\:{i}^{th}$ bat value at the $\:{j}^{th}$ location in $\:(t+1)$ cycle, $\:j=\text{1,2},3,...,m$. rand(0,1) indicates a random value in the range [0,1]. If $\:R\left(\text{0,1}\right)\:>{r}_{i}$, the next iteration’s velocity is updated using Eq. (5), and the updated position is subsequently converted into binary using the following equations^17^.

23 $$\:s\left({Vx}_{i}^{j}\right)=\frac{1}{1+{e}^{-{Vx}_{i}^{j}}}$$

24 $$\:{x}_{i}^{j}\left(t+1\right)=\left\{\begin{array}{c}0\:\:\:\:\:\:\:\:\:\:\:\:\:\:\:\:\:\:\:\:\:\:\:\:\:\:if\:\:\:rand\left(\text{0,1}\right)\ge\:s\left({Vx}_{i}^{j}\right)\\\:\:\\\:\:1\:\:\:\:\:\:\:\:\:\:\:\:\:\:\:\:\:\:\:\:\:\:\:\:\:\:\:\:otherwise\:\:\:\:\:\:\:\:\:\:\:\:\:\:\:\:\:\:\:\:\:\:\:\:\end{array}\right.\:$$

Then, evaluate all solution using Eq. (12). Furthermore, if $\:\left(R\right(\text{0,1})<\:Ai)\:$ and $\:\left(F\right({x}_{i})\:<F({{x}_{g}}^{*}\left)\right)$ then the loudness and emission rate are adjusted according to Eqs. (18) and (19), and the new solution is accepted. The calculations are then repeated until the specified number of generations is reached. Ultimately, the superior bat of the entire population $\:{x}_{g}$ is generated, and the algorithm terminates. All features contributed by 1 are paramount for obesity prediction. Table 2 illustrated the selected features by EC-QBA and their clinical relevance to obesity. Additionally, Algorithm 1 delineates the sequential procedures of EC-QBA.

**Table 2:** The selected features by EC-QBA and their clinical relevance to obesity.

Selected features	Clinical relevance
Weight	A direct assessment of body mass index, it is essential for categorizing BMI and obesity.
Gender	Affects fat distribution and hormonal factors associated with obesity
Age	It affects metabolism, fat distribution, and the risk of obesity in the long term.
family_history_with_overweight	Genetic and environmental susceptibility to obesity
FAVC	Indicates dietary quality; increased consumption of high-calorie foods elevates the risk of obesity.
FAF	A significant modifiable factor; consistent physical activity aids in the prevention of weight gain.
CAEC	Snacking behaviors that can elevate overall caloric consumption and contribute to obesity.

**Algorithm 1:**
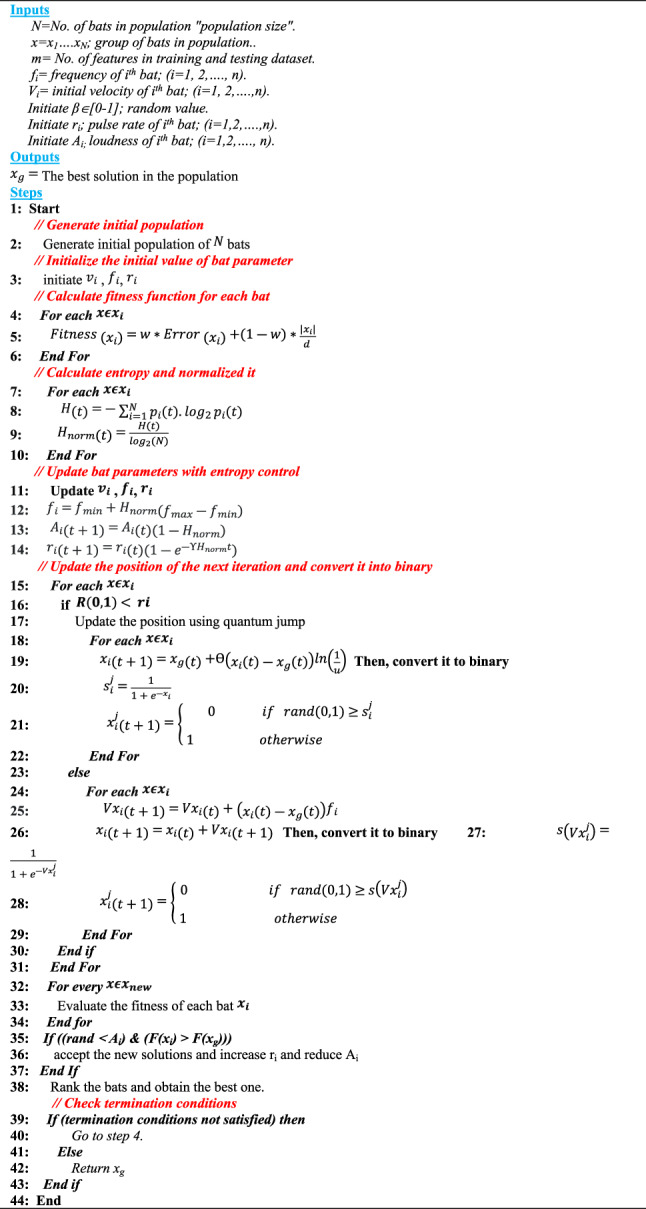
The sequential procedures of EC-QBA.

In conclusion, EC-QBA is a new feature selection methodology that enhances traditional BA by integrating two principal improvements:

(i) Entropy controlled of BA parameters (i.e., frequency, loudness, and pulse rate).

- Shannon entropy quantifies the diversity or dispersion of the existing solution set.
- High entropy signifies elevated diversity and amplifies exploitation.
- Low entropy signifies early convergence towards a local optimum, thereby facilitating exploration.
- This adaptation enables the algorithm to judiciously manage the equilibrium between exploration and exploitation.

(ii) A local search mechanism inspired by quantum mechanics: EC-QBA adds a quantum search mechanism that updates each bat’s location using the principle of quantum probabilities instead of traditional updates that leads to:

- Faster convergence toward optimal solutions.
- Selection of higher-quality feature sets and improved classification accuracy.
- Flexibility in handling complex, high-dimensional data spaces.
- Improved performance of models later used in predicting obesity levels.

#### Illustrative example

**Fig. 7:**
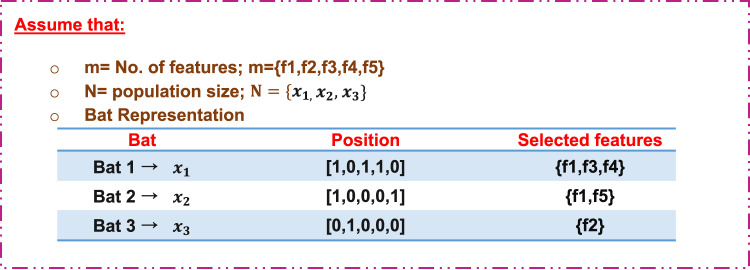
An illustrative example of bats representation.

**Fig. 8:**
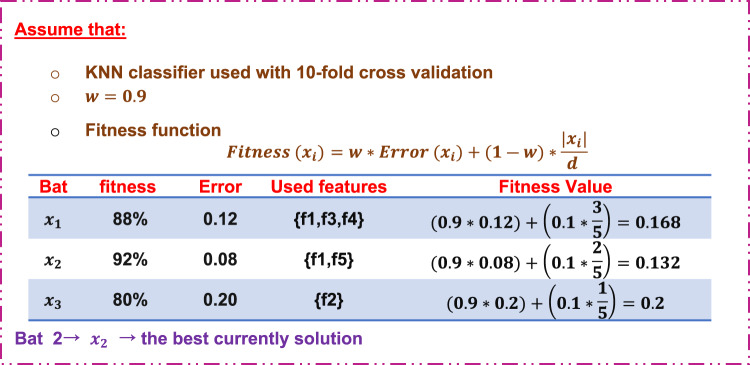
An illustrative example of fitness function calculation.

**Fig. 9:**
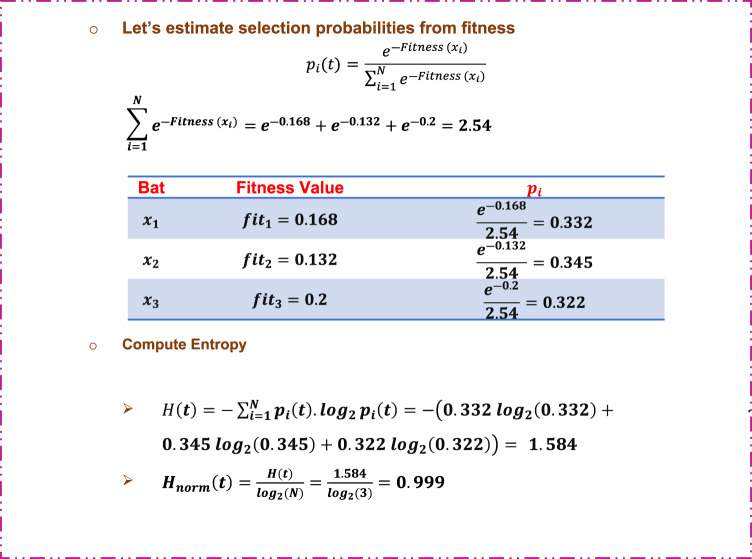
An illustrative example of normalized entropy calculation.

**Fig. 10:**
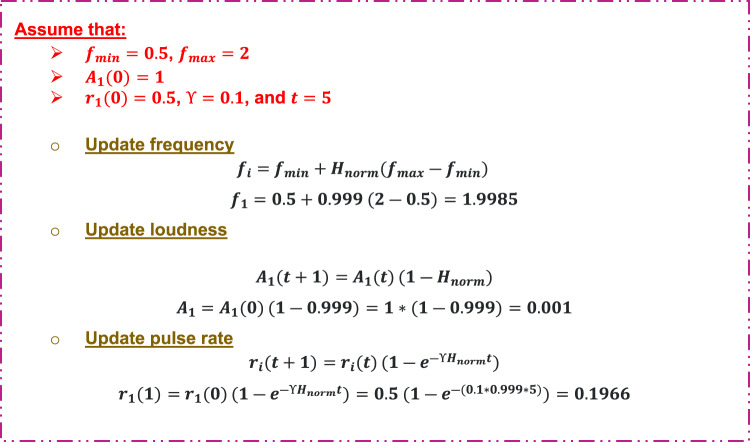
An illustrative example of updating bat parameters.

**Fig. 11:**
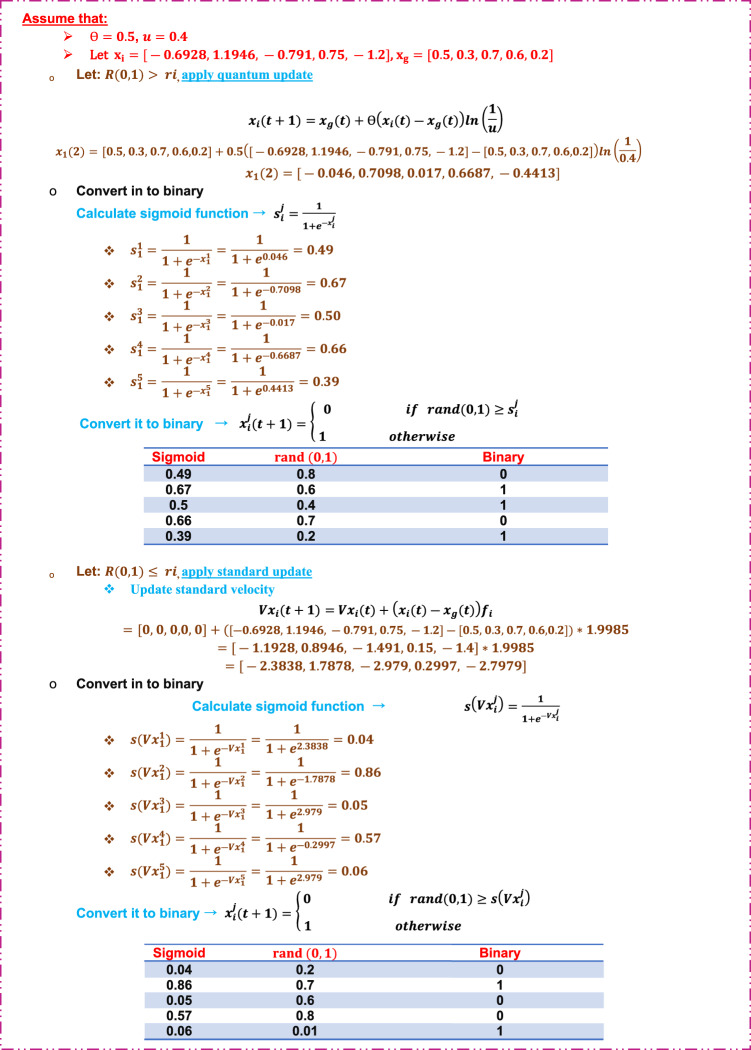
An illustrative example of updating bats position.

To illustrate the idea of the proposed EC-QBA, through this subsection a numerical example will be discussed to illustrate the sequential steps of it as shown in (Figs. 7, 8, 9, 10 and 11).

- Step 1: Bat representation.

Figure 7 shows the generation of initial bat population.

- Step 2: Fitness function calculation.

Figure 8 illustrates how to calculate fitness values using KNN.

- Step 3: Normalized entropy calculation (Shannon entropy).

Figure 9 illustrates how to compute entropy and normalized entropy.

- Step 4: Adapt parameters with entropy.

Figure 10 illustrates how to use normalized entropy to update frequency, loudness and pulse rate.

- Step 5: Update bat position.

Figure 11 illustrates how to update bat position for the next iteration and how to perform quantum jump. Additionally, illustrates how to convert the updated position into binary.

### Obesity risk prediction (ORP)

Obesity risk prediction (ORP) is the final stage of our framework, where the final decision is made. In this stage, several ML models are used to make the prediction process, and the decision is made by the majority vote. These models are Linear Regression (LR), LightGBM (LGBM), XGBoost (XGB), AdaBoost, Multi-Layer Perceptron (MLP), KNN, and SVM^40,41^:

- Linear regression (LR): LR served as an efficient and basic model, well-suited for tasks involving binary and multiclass classification^42^.
- LightGBM (LGBM): LGBM, renowned for its rapidity and precision, was employed to effectively manage extensive data sets by constructing distinctive vertical decision trees^43^.
- XGBoost (XGB): The versatile XGB was included due to its capacity to manage intricate data relationships^44^.
- AdaBoost: It is an ensemble technique that focuses on integrating weak learners and utilizes an iterative process to improve classification precision^45^.
- Multi-layer perceptron (MLP): MLP neural network was used to handle complicated, nonlinear data^46^.
- K-nearest neighbors (KNN): KNN is proposed as an intuitive methodology for classification, taking into account the predominant common type among the points of data’s immediate neighbors^47^.
- Support vector machine (SVM): SVM was utilized for its adaptability in managing high-dimensional data and both linear and nonlinear classification tasks^47^.

Table 3 illustrates why these specific ML algorithms were chosen and how they contribute to the majority voting decision in the prediction stage.

**Table 3:** The role in decision of each ML algorithm.

Algorithm	Strength	Role in decision
LR	Simple to implement, and fast	Provides a strong linear baseline
LGBM	Fast, accurate, works efficiently on big data	Very powerful model that supports accuracy.
XGB	Very powerful, and handles missing data	A very accurate model that supports performance.
AdaBoost	Reduces bias, and good with noisy data	Adds bias correction and is good at helping weak learners get better.
MLP	Handles complex non-linear patterns	Adds deep learning capability to the group
KNN	Simple, reflects the local structure of the data	Enhances diversity in location-specific predictions
SVM	Efficient in high-dimensional spaces.	Brings regular decision boundaries to the group.

Hyperparameter optimization is crucial for ML models, particularly for neural networks, which are generally considered black box models. It cannot interfere with model training because adjusting hyperparameters prior to the formal execution of the model becomes a crucial method for enhancing model accuracy^48^. The preliminary manual tuning, succeeded by the evolution of grid and random search, was demonstrated to be laborious and ineffective. Subsequently, a variety of methods for the automatic tuning of parameters were developed, which were based on the principles of efficiency and accuracy. Bayesian optimization is a technique that involves the use of a model to determine the value that reduces the objective function, thereby minimizing the function. It is highly efficient and time-effective, as it employs the results of previous evaluations to evaluate the subsequent set of hyperparameters^48,49^.

**Fig. 12:**
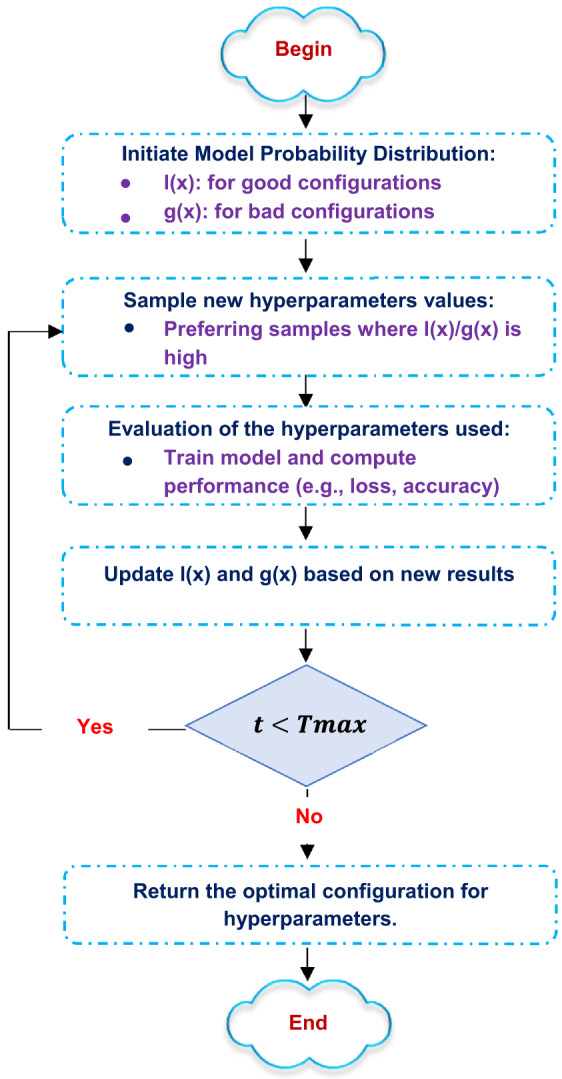
The steps of TPE for hyperparameters optimization.

Tree parsen estimator (TPE) is an efficient and robust methodology for hyperparameter optimization. It is a probabilistic model-based optimization algorithm that quickly searches the hyperparameter search space for the best settings for ML models. Its workflow can be summarized as follows:

(i) Modeling probability distributions: TPE models two probability distributions:

- One for promising hyperparameter configurations (exploitation).
- One for less promising configurations (exploration).

(ii) Sampling: New hyperparameter values are sampled in a way that favors the promising regions while still allowing for exploration of other areas in the search space.
(iii) Objective function evaluation: The sampled hyperparameters are employed to train the model, and their efficacy is assessed using a predetermined objective function (e.g., accuracy, loss).
(iv) Updating distributions: Based on the observed performance, TPE updates both distributions to reflect new insights, allocating more sampling probability to better-performing regions.
(v) Iterative search: This process is repeated iteratively, allowing TPE to refine its probabilistic model and converge towards optimal hyperparameter configurations.
(vi) Final selection: Upon completion of the specified number of iterations, TPE yields the optimal hyperparameter configuration, suitable for training the final model. Figure 12 illustrates TPE principles.

**Fig. 13:**
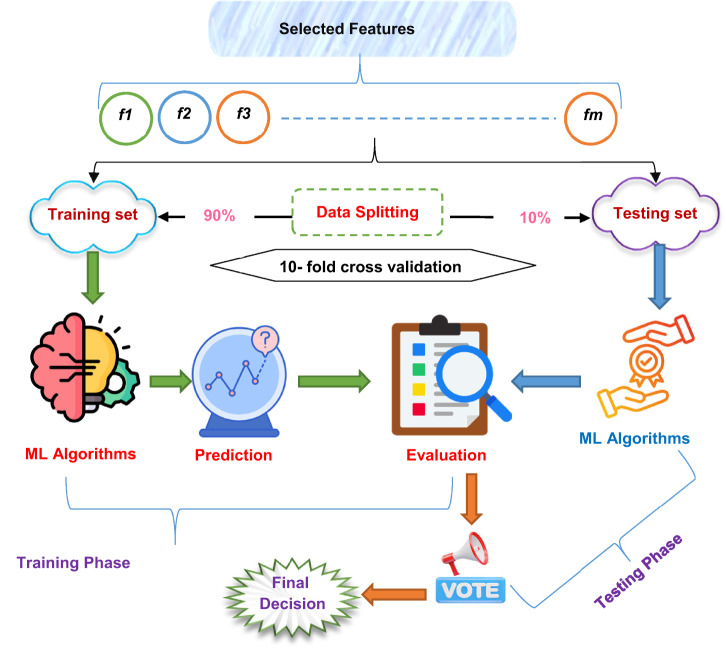
The framework of ORP stage.

**Fig. 14:**
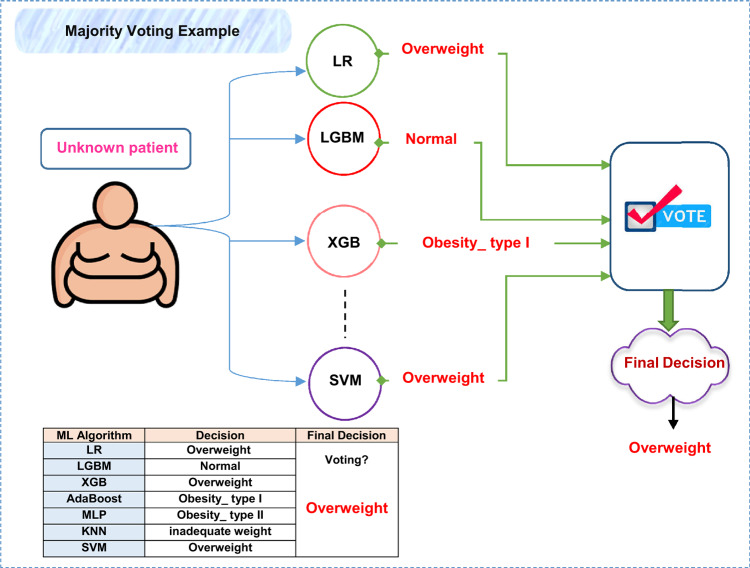
An illustrative example of ORP process.

Finally, the selected features are fed to these optimized models as illustrated in (Fig. 13). As shown in Fig. 13, the training process is performed, keeping the previous stages in these models. Then, the testing process is applied to determine the final decision according to the model that achieves the highest accuracy. Figure 14 illustrates the idea of majority voting.

## Experimental results

In this part, we will assess our framework. Our framework is implemented through three stages: preprocessing stage (PS), feature stage (FS), and obesity risk prediction (ORP). Several steps are taken to preprocess the input data through PS, including the filling of missing values, feature encoding, the removal of outliers, and normalization. Next, the preprocessed features are sent to FS to identify the paramount features utilizing the proposed entropy controlled-quantum bat algorithm (EC-QBA). EC-QBA is a new feature selection methodology that is based on BA with two variations. The first is updating BA parameters (i.e., frequency, loudness, and pulse rate) using normalized entropy. The second variation involves updating the new position through a quantum mechanism. Finally, these selected features are sent to several ML algorithms, and the decision is made by the majority vote. Our framework is a viable way to predict obesity risk because it combines smart feature selection, deep representation learning, and attention-driven interpretability. The execution of the suggested system is predicated on detailed information on individuals, including essential characteristics such as gender, age, height, weight, familial predisposition to obesity, dietary practices, physical activity, mode of transportation, and associated obesity levels^50^. The prediction model is evaluated using cross-validation. The dataset is divided into ten equal parts, with one part serving as the test set and the other nine as training sets. Ten-fold cross-validation is used in this paper to achieve this. Consequently, 18,682 patients (90%) are involved in the training phase, while 2076 patients (10%) are involved in the testing phase. All experiments were conducted on a system with an Intel Core i7 CPU, 16 GB RAM, and Python 3.10. Table 4 presents the parameters utilized and the values implemented.

**Table 4:** The parameters used along with their corresponding values.

Parameter	Description	Algorithm	Applied value
N	No. of features	–	16
$\:Tmax$	Maximum number of iterations		50
w	Constant	For fitness function	0.99
C	–	LR	logNormal (µ = 0, σ = 1.)
Solver	–		lbfgs
max_depth	Maximum depth of the tree	LGBM	Choice from range (1, # Features + 1)
n_estimators	Number of boosting iterations (trees).		100
$\:r$	Learning rate		0.05
Subsample	Fraction of rows to use per boosting round	XGB	Subsample (Uniform(0.1, 1)),
n_estimators	Number of boosting rounds (trees)		100
max_depth	Maximum depth of each tree.		6
$\:r$	Learning rate		0.01
n_estimators	Number of boosting rounds (i.e., number of weak learners).	AdaBoost	100
$\:r$	Learning rate		0.01
w	weights	KNN	Choice from [“Uniform”, “Distance”]
Algorithm	Algorithm to compute neighbors		Choice from [“Ball Tree”, “KDTree”, “Brute”]
n_neighbors	No. of neigbours		10
Metric	Distance metric to use		‘Minkowski’
Activation	Activation function	MLP	‘relu’
Solver	Optimization Algorithm		‘adam’
Learning_rate_init	Initial Learning Rate		0.001
Hidden_layer_size	No. of neurons in hidden layer		(100,50)
C	Regularization parameter	SVM	2
kernel	Specifies the kernel type		‘rbf’
gamma	Kernel coefficient for ‘rbf’		‘auto’
a	Control parameter	GWO	Linearly decrease from 2 to 0 over repetitions
r_1_, r_2_	Random vectors		r_1_, r_2_ \in [0,1]
Ps	Probability of selection	GA	0.9
Pc	Probability of crossover		0.9
Pm	Probability of mutation		0.1
w	Inertia weight	PSO	1.1
c1	The cognitive acceleration		2
r1	Uniformly distributed random number		0.6
r2			

### Dataset description

**Fig. 15:**
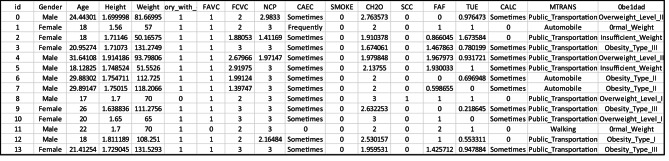
A sample of the used dataset.

**Fig. 16:**
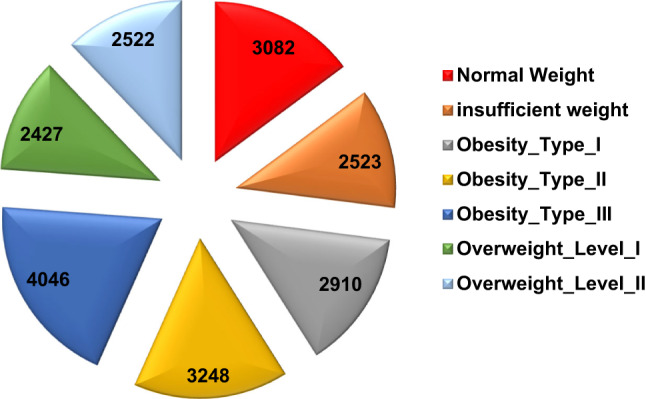
The distribution of weights type.

**Fig. 17:**
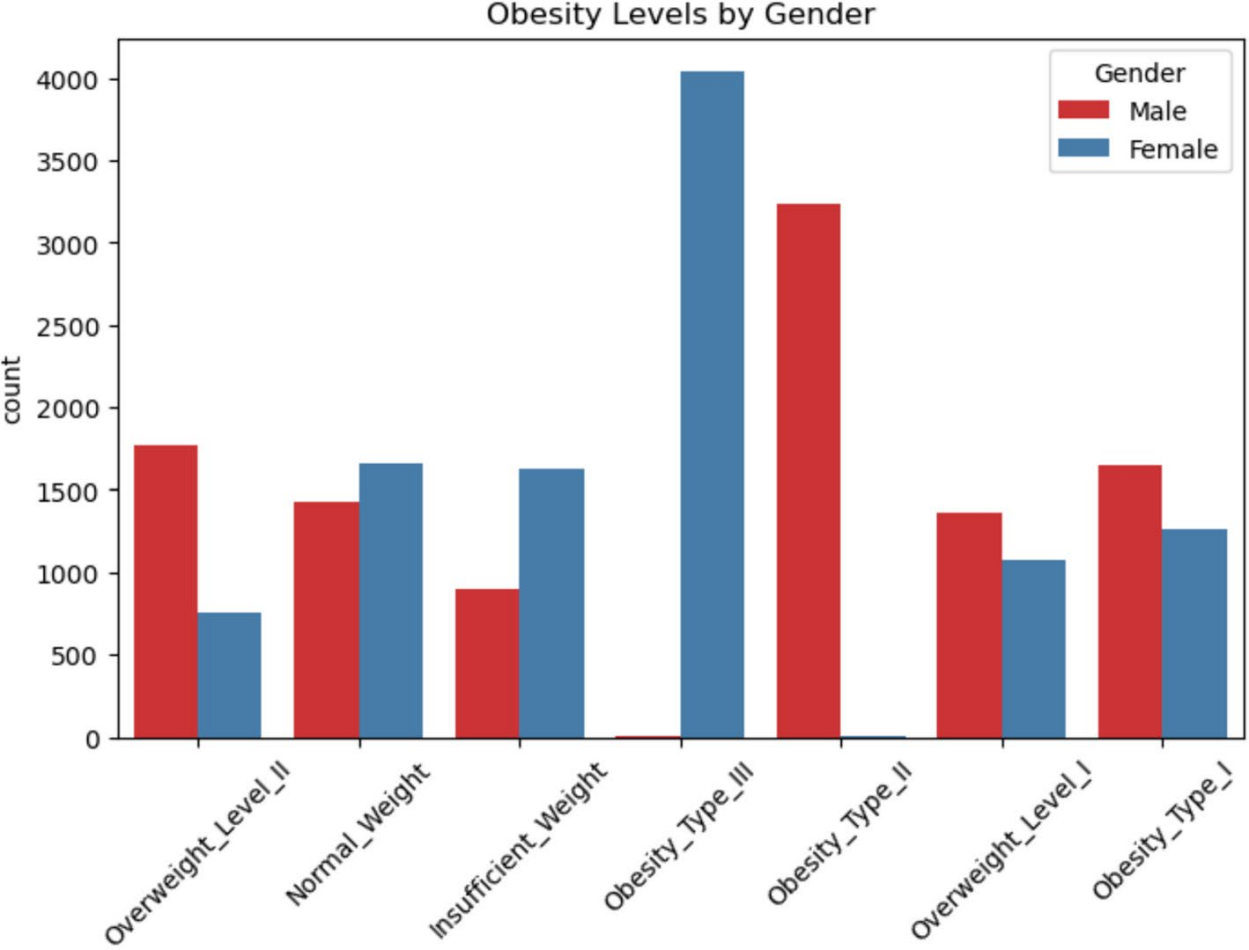
The distribution of the used dataset according to gender.

In this study, we used a dataset that was retrieved from Kaggle^50^ consisting of 20,758 cases, each with 16 features related to demographic, behavioral, and lifestyle factors. These features include gender, age, height, weight, family_history_with_overweight, Frequent consumption of high-caloric food (FAVC), Frequency of consumption of vegetables (FCVC), Number of main meals (NCP), Consumption of food between meals (CAEC), smoke, Daily water consumption (CH2O), Caloric beverages consumption (SCC), Physical activity frequency (FAF), Time spent using technological devices (TUE), Consumption of alcohol (CALC), Mode of transportation (MTRANS). Table 5 provides an overview of the structure of the data set. Figure 15 shows an example of a sample from the used dataset, while Figs. 16 and 17 show the distribution of obesity levels and gender representation, respectively.

**Table 5:** The distribution of the used dataset according to gender.

Obesity type	Male	Female	Total
Normal weight	1422	1660	3082
insufficient weight	902	1621	2523
Obesity_Type_I	1643	1267	2910
Obesity_Type_II	3240	8	3248
Obesity_Type_III	5	4041	4046
Overweight_Level_I	1357	1070	2427
Overweight_Level_II	755	1767	2522
Total	9324	11,434	20,758

**Fig. 18:**
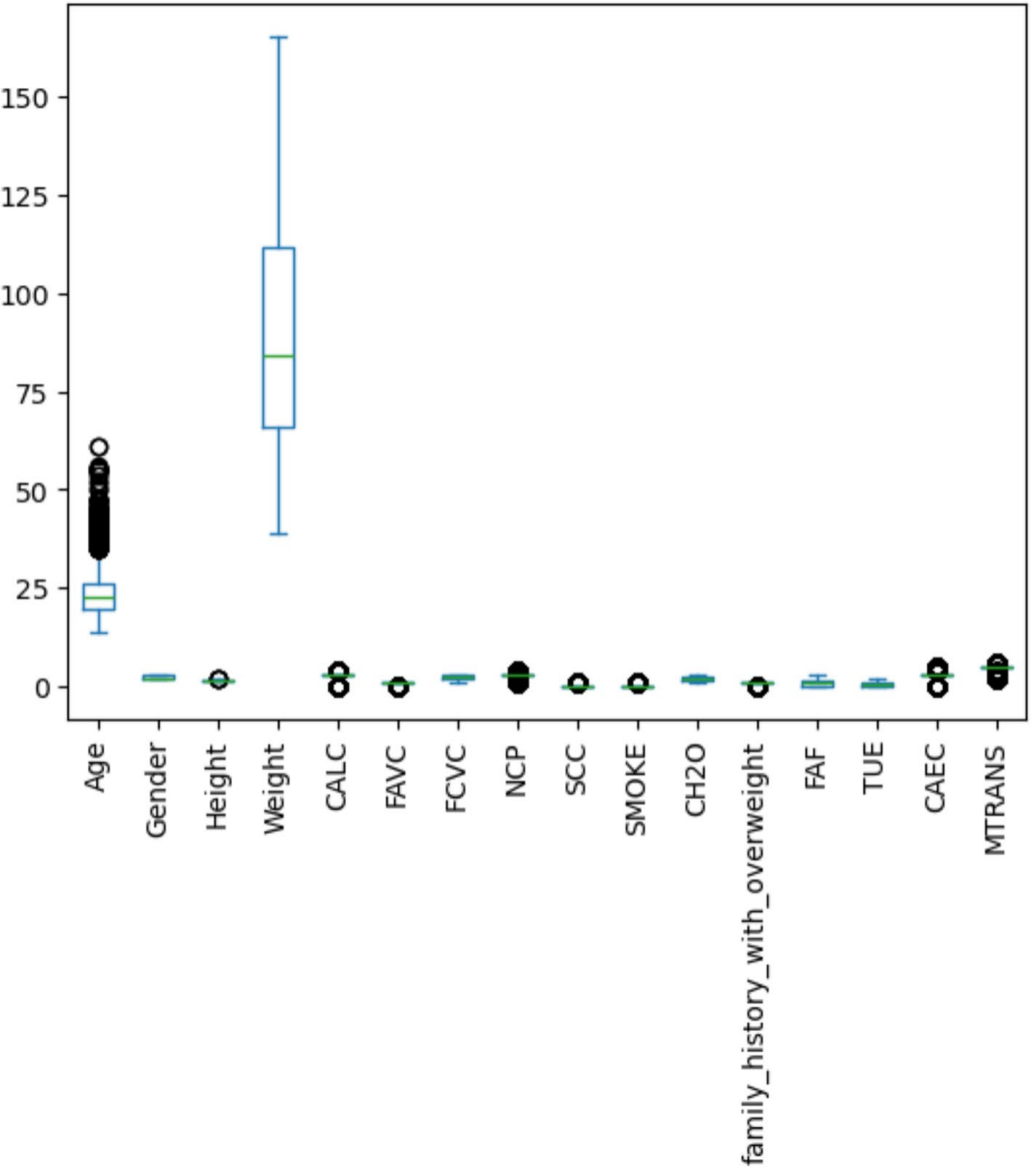
The boxplot of features in the used dataset.

**Fig. 19:**
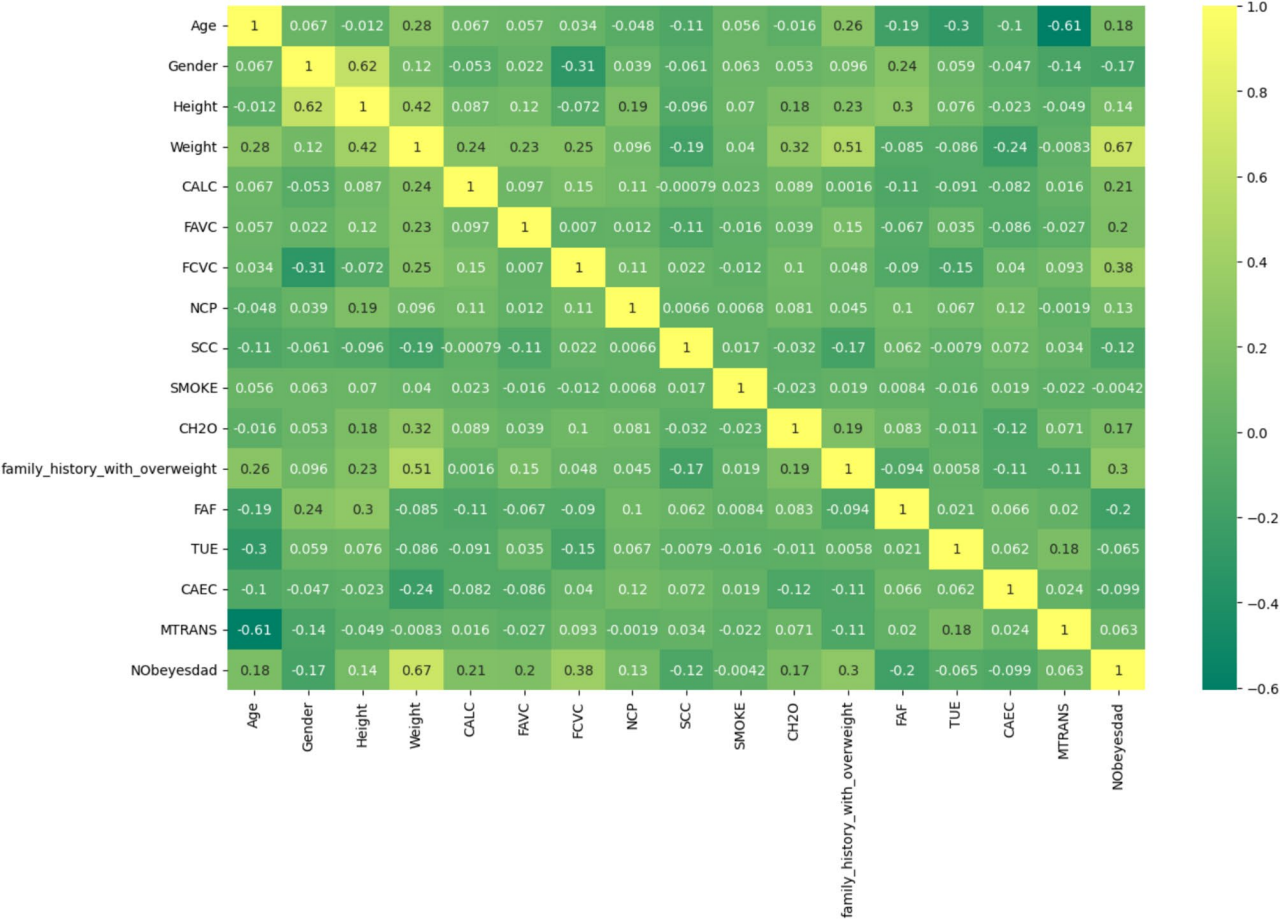
A heat map showing the overall correlation between all variables and the correlation between features and obesity type.

Table 6 also includes the range of values, including minimum and maximum, average, and standard deviation. At its lowest point, the recorded value indicates the dataset’s lowest magnitude. The highest recorded value, or maximum value, indicates the largest observed value. As a measure of the typical value, the mean (or average) is calculated by taking the mathematical mean of all the data points in a set. Data variability can be better understood by calculating the standard deviation, which measures the dispersion of data points relative to the mean. To better evaluate and understand the experimental results, the variable or parameter under analysis has its extreme values, variability, and mean value clarified. Figure 18 displays the features from the dataset in a boxplot. To further evaluate the association between each characteristic and the type of obesity, correlation analysis was also performed. Figure 19 shows the results of the correlation analysis that was used to determine if the relationship between each pair of variables was mostly positive or negative.

**Table 6:** The statistical analysis of the used dataset.

Feature	Count	Mean	Std	Min	25%	50%	75%	Max
id	20,758	10378.5	5992.463	0	5189.25	10378.5	15567.75	20,757
Gender	20,758	0.497929	0.500008	0	0	0	1	1
Age	20,758	742.1278	472.3441	0	356	761	1152	1702
Height	20,758	861.2385	510.8984	0	420	822	1287	1832
Weight	20,758	850.8906	579.5313	0	333	718	1391	1978
family_history_with_overweight	20,758	0.819636	0.3845	0	1	1	1	1
FAVC	20,758	0.914443	0.279716	0	1	1	1	1
FCVC	20,758	557.0513	339.033	0	233	501	933	933
NCP	20,758	465.2157	185.0616	0	542	542	542	688
CAEC	20,758	2.794537	0.538479	0	3	3	3	3
SMOKE	20,758	0.011803	0.108	0	0	0	0	1
CH2O	20,758	715.7404	457.8653	0	476	650	1091	1505
SCC	20,758	0.033096	0.178891	0	0	0	0	1
FAF	20,758	595.7702	465.9449	0	15	668	995	1359
TUE	20,758	532.3176	468.6583	0	0	532	969	1296
CALC	20,758	1.477069	0.864267	0	1	2	2	2
MTRANS	20,758	2.506841	1.14873	0	3	3	3	4
0be1dad	20,758	2.964544	1.928104	0	1	3	4	6

**Fig. 20:**
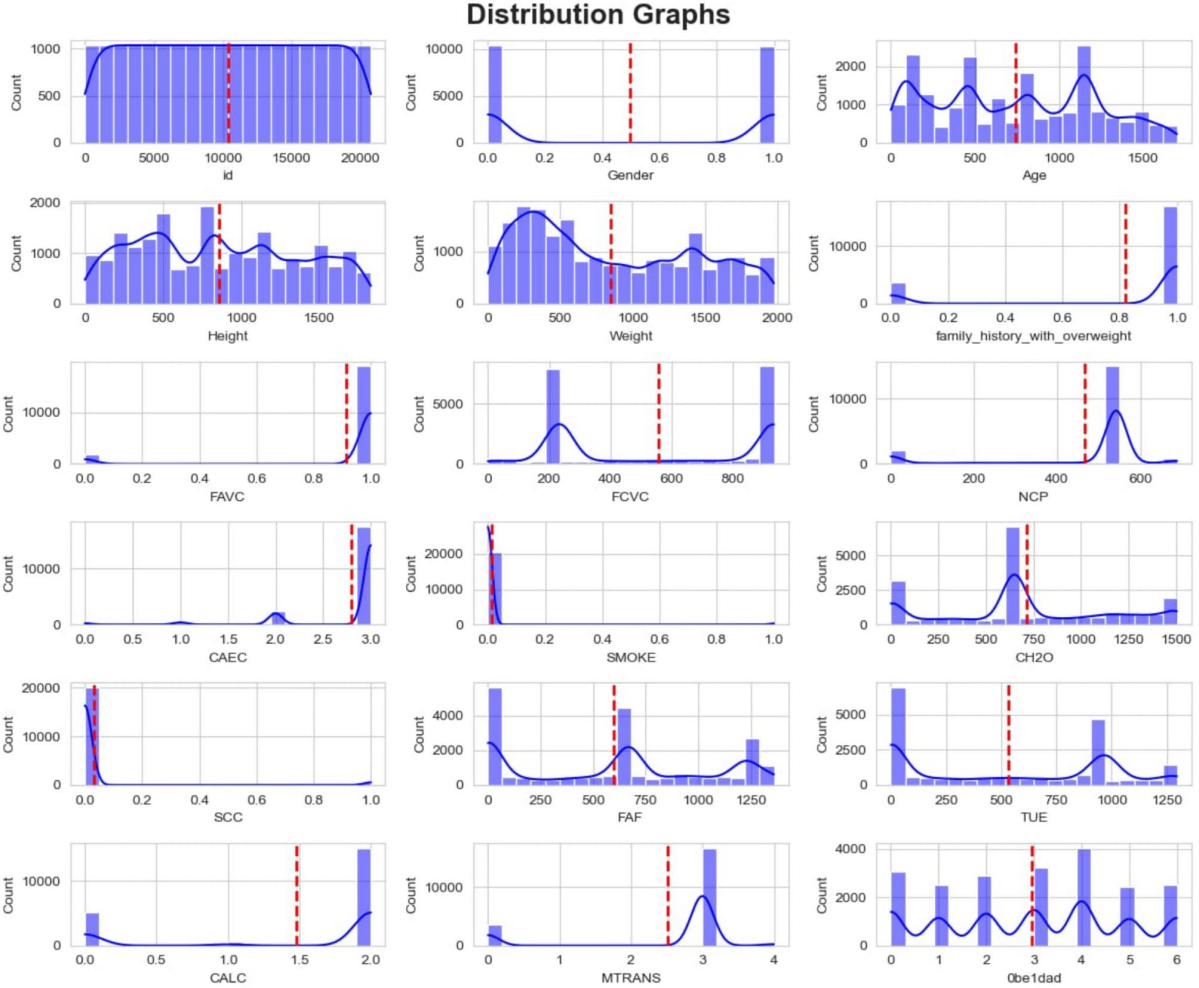
Histogram of features of the used dataset.

**Fig. 21:**
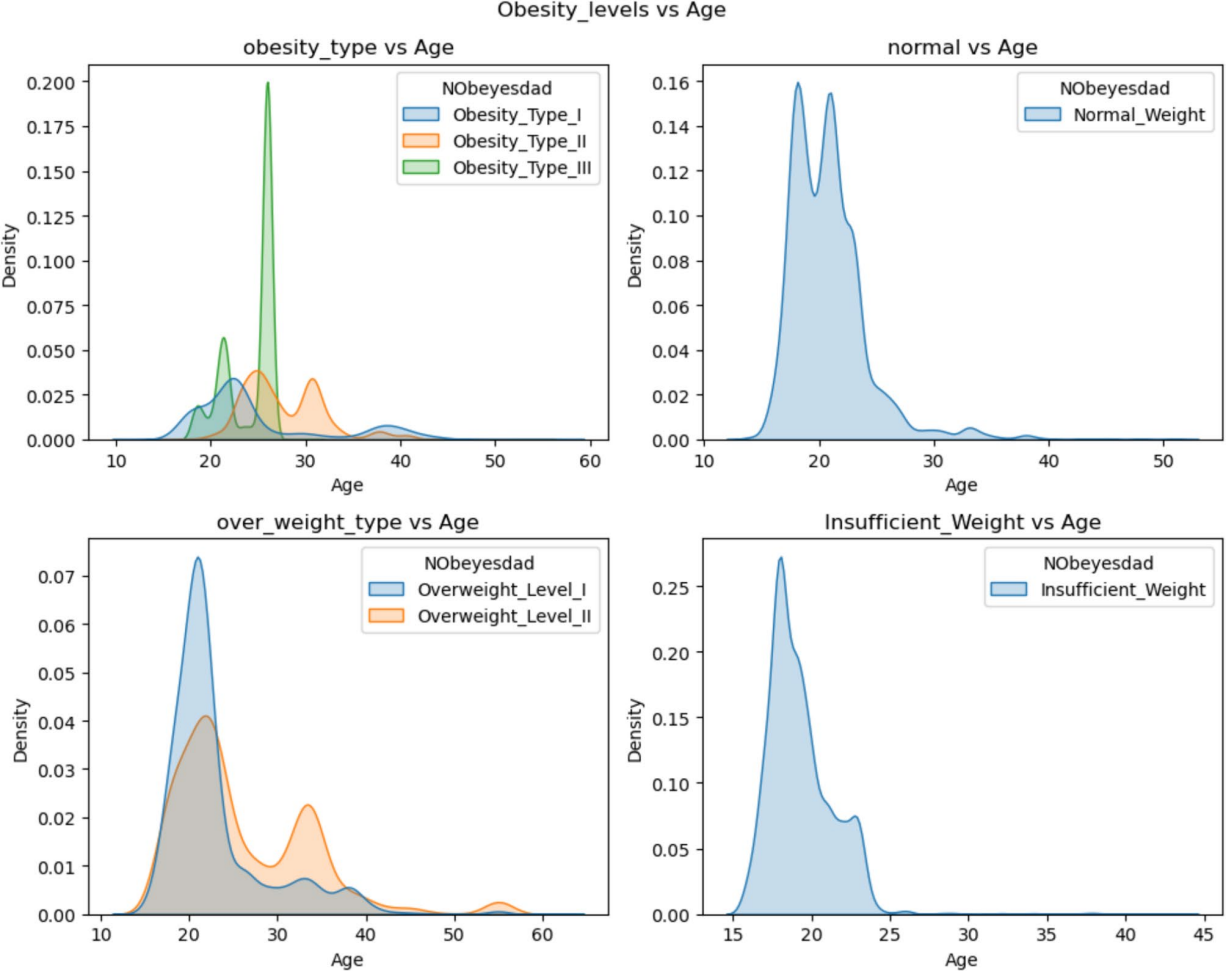
Obesity level vs. age.

Additionally, Fig. 20 displays histograms that are normally distributed, categorizing all features within the specified value range. The x-axis denotes the nature or type of each attribute, whereas the y-axis indicates the corresponding feature value. Moreover, Fig. 21 indicates the obesity level with respect to age.

### Evaluation metrics

In the upcoming tests, evaluation metrics like precision, sensitivity, accuracy, and F-measure will be calculated. The following equations can be used to calculate these metrics^51^:

25 $$\:Precision=\frac{{T}_{P}}{({T}_{P}\:+\:{F}_{P})}$$

26 $$\:Sensitivity=\frac{{T}_{P}}{({T}_{P}\:+\:{F}_{N})}$$

27 $$\:Accuracy=\frac{({T}_{P}\:+\:{T}_{N})\:}{\:({T}_{P}\:+\:{T}_{N}+\:{F}_{P}\:+\:{F}_{N})}\:$$

28 $$\:F-measure=\frac{2*Recall*Precision}{\left(Recall+Precision\right)}$$

Where $\:{T}_{P}$ refers to true positive, which signifies the ratio of anticipated positive samples. $\:{T}_{N}$ is a true negative, signifying the anticipated quantity of non-positive samples. $\:{F}_{P}$ is a false positive, representing the ratio of expected positive instances within negative samples. $\:{F}_{N}$ is a false positive, denoting the number of positive samples expected to be misclassified as negative.

### Testing the proposed entropy controlled-quantum Bat algorithm (EC-QBA)

**Fig. 22:**
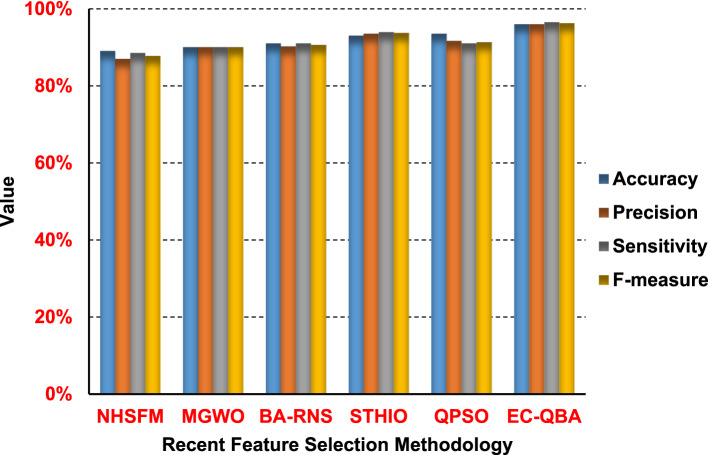
Comparison between the most recent feature selection methods and EC-QBA.

In this subsection, the proposed entropy controlled-quantum bat algorithm (EC-QBA) will be evaluated. To prove the effectiveness of the proposed EC-QBA, we compared it with the most recent feature selection method using NB as a base classifier. These methods are the new hybrid feature selection method (NHFSM)^52^, modified grey wolf optimization (MGWO)^53^, Bat Algorithm with the residue number system (BA-RNS)^54^, sheep-tuna halcyon integrated optimization (STHIO)^55^, and quantum particle swarm (QPSO)^56^. Results were shown in (Fig. 22).

As shown in Fig. 22, the proposed EC-QBA outperforms the other feature selection methodology with regard to accuracy, precision, sensitivity, and F-measure. It offers 96% accuracy, 96% precision, 96.5% sensitivity, and 96.25% F-measure. The worst performance was achieved by NHFSM, which reported 89% accuracy, 87% precision, 88.5% sensitivity, and 87.74% F-measure; MGWO followed with 90% accuracy, 90% precision, 90% sensitivity, and 90% F-measure. In conclusion, the best performance is obtained by the proposed EC-QBA, as it is based on a very effective strategy of adjusting BA parameters; this strategy uses Shannon entropy. Additionally, EC-QBA uses a quantum mechanism to update the BA solution in local search. Consequently, it avoids BA’s drawbacks.

In addition, to further evaluate the efficiency of the proposed EC-QBA, we fed the selected features into Logistic Regression (LR) as a baseline model to highlight EC-QBA’s added value. According to the obtained results, it introduces an accuracy of 78% without using EC-QBA. While when using EC-QBA, it introduces 86% with a 9% improvement. This proves the value that EC-QBA added to any ML model, even one.

Furthermore, We assessed the computational cost of the EC-QBA algorithm regarding execution duration and memory utilization, juxtaposing it with various conventional feature selection algorithms, including Recursive Feature Elimination (RFE) and Mutual Information (MI). Table 7 illustrates that the EC-QBA algorithm exhibits a longer execution time with an average value of 13.85 s and, with 136.7 MB, higher memory consumption attributable to its implementation of a transaction control mechanism utilizing entropy and a quantum update mechanism. Notwithstanding the elevated computational cost, the superior feature selection accuracy achieved through EC-QBA markedly improves the predictive model’s performance, rendering it justifiable in scenarios necessitating high precision.

**Table 7:** Comparison between EC-QBA and other competitors in terms of computational cost.

Feature selection method	Average run time (Second)	Standard deviation	Memory usage (MB)
RFE	3.35	0.23	79.5
MI	1.97	0.04	64.1
EC-QBA	13.85	0.52	136.7

### Comparison between EC-QBA and standard approaches

In this section, the efficacy of the proposed EC-QBA is demonstrated by comparing several methods for selecting features using LR classifier, which serves as the base classifier. These approaches were recursive feature elimination (RFE), principal component analysis (PCA), genetic algorithm, grey wolf optimization (GWO) and particle swarm optimization (PSO). Results were shown in (Table 8).

**Table 8:** Comparison between EC-QBA and standard approaches based on LR.

Method	Accuracy %	Precision %	Sensitivity %	F-measure %
RFE	75.0 ± 1.5	72.0 ± 1.2	70.0 ± 1.0	70.98 ± 1.3
PCA	78.0 ± 1.4	73.5 ± 1.1	71.9 ± 1.2	72.69 ± 1.2
GA	79.5 ± 1.3	74.0 ± 1.0	72.8 ± 1.1	73.39 ± 1.3
GWO	80.5 ± 1.2	75.0 ± 1.2	73.0 ± 1.1	73.98 ± 1.0
PSO	82.0 ± 1.1	79.0 ± 1.3	78.0 ± 1.2	78.49 ± 1.1
EC-QBA	86.0 ± 1.0	84.1 ± 0.9	83.8 ± 1.0	83.9 ± 0.9

As shown in Table 8, the proposed EC-QBA outperformed the other standard algorithms in terms of accuracy, precision, sensitivity, and F-measure. It introduces 86.0% ± 1.0 accuracy, 84.1%± 0.9 precision, 83.8%±1.0 sensitivity, and 83.9%± 0.9 F-measure. The lowest performance was obtained by RFE as it introduced 75% ± 1.5 accuracy, 72%± 1.2 precision, 70%± 1.0 sensitivity, and 70.98% ± 1.3 F-measure. According to the Table 8, the proposed EC-QBA is more effective in selecting the most important features than other traditional methods when LR used as a base classifier.

### Ablation studies: evaluating the impact of key components

In this section, we will test the effect of each of the proposed EC-QBA components to demonstrate their effectiveness. To achieve this goal, first, exclude entropy control and quantum jump, and only use Binary Bat Algorithm (FS_modeL1). After that, we exclude the use of quantum jumps and remain only with the use of entropy control (FS_modeL2). Finally, we exclude the use of entropy while updating the bat frequency, loudness, and pulse rate in a normal manner, leaving only quantum jump (FS_modeL3) as presented in (Table 9). Results are shown in (Table 10).

**Table 9:** The structural components within the EC-QBA.

Model variation	Entropy controlled	Quantum update	Prediction model
BBA	x	x	FS_modeL1
BA + entropy	✓	x	FS_modeL2
BA + quantum	x	✓	FS_modeL3
EC-QBA (Full)	✓	✓	ObeRisk

**Table 10:** Evaluating the impact of key components of EC-QBA.

Model	Accuracy %	Precision %	Sensitivity %	F-measure %
FS_modeL1	89.0 ± 1.3	75.0 ± 1.5	74.6 ± 1.2	74.8 ± 1.1
FS_modeL2	94.3 ± 0.8	87.0 ± 1.0	87.2 ± 0.9	87.1 ± 0.9
FS_modeL3	92.0 ± 1.0	86.0 ± 1.1	86.4 ± 1.0	86.2 ± 1.1
ObeRisk	97.1 ± 0.4	95.7 ± 0.5	95.5 ± 0.4	9.6 ± 0.4

Table 9 demonstrates that ablation studies were conducted to evaluate components that substantially influence accuracy and reliability in selecting the most important features. Each experiment isolates a fundamental element of our proposed method. As shown in Table 10, when using traditional BBA for feature selection (FS_modeL1), it introduces 89.0% ± 1.3 accuracy, 75.0% ± 1.5 precision, 74.6% ± 1.2 sensitivity, and 74.8% ± 1.1 F-measure, which are the worst results. While when excluding the quantum jump in updating the position of the bat in the next iteration in local search, it introduces 94.3% ± 0.8 accuracy, 87.0% ± 1.0 precision, 87.2% ± 0.9 sensitivity, and 87.1% ± 0.9 F-measure. Additionally, when excluding entropy control (FS_modeL3), it introduces 92.0% ± 1.0 accuracy, 86.0% ± 1.1 precision, 86.4% ± 1.0 sensitivity, and 86.2% ± 1.1 F-measure. Finally, the obtained results illustrate the ability of the proposed EC-QBA to identify the most critical features.

### Testing the proposed oberisk

Through this subsection, our framework will be tested and evaluated to select the most efficient algorithm to complete the proposed model. In fact, ObeRisk consists of three main parts; the first part (i.e., PS) is responsible for cleaning the used dataset and making it in suitable form for the next part (i.e., FS). In FS, the proposed EC-QBA is employed to select the most significant and beneficial features. Finally, these effective features are sent to several ML algorithms, and the decision is made by the majority vote. These ML algorithms are LR, KNN, SVM, LGBM, XGB, MLP, and AdaBoost. Each model was trained using 10-fold cross validation, and results were shown in (Table 11).

**Table 11:** Comparison between using single classifier and ObeRisk.

Model	Accuracy (%)	Precision (%)	Sensitivity (%)	F-measure (%)
LR	86.0 ± 1.2	84.1 ± 1.3	83.8 ± 1.1	83.9 ± 1.2
KNN	73.8 ± 2.4	73.8 ± 2.4	72.1 ± 2.5	72.0 ± 2.3
SVM	85.7 ± 1.1	83.9 ± 1.0	83.6 ± 1.1	83.7 ± 1.1
LGBM	90.1 ± 0.9	89.3 ± 0.8	89.2 ± 0.9	89.1 ± 0.9
XGB	91.2 ± 0.7	90.1 ± 0.7	90.3 ± 0.6	90.2 ± 0.7
MLP	85.3 ± 1.5	82.8 ± 1.6	83.1 ± 1.4	83.0 ± 1.5
AdaBoost	74.6 ± 2.2	73.9 ± 2.1	73.4 ± 2.3	73.5 ± 2.2
ObeRisk	97.1 ± 0.4	95.7 ± 0.5	95.5 ± 0.4	95.6 ± 0.4

Among all compared models, ObeRisk achieved the best overall performance with an accuracy value of 97.1% ± 0.4, significantly outperforming traditional models like LR with an accuracy value of 86.0% ± 1.2 and KNN with an accuracy value of 73.8 ± 2.4. Other algorithms that performed strongly included XGB with an accuracy value of 91.2% ± 0.7 and LGBM with an accuracy of 90.1% ± 0.9, although they still trailed ObeRisk by a significant margin. Consequently, depending on several ML algorithms is more important than using a single model. We utilize our majority vote-based framework to enhance classification results by integrating basic models. However, it also presents high computational complexity.

**Fig. 23:**
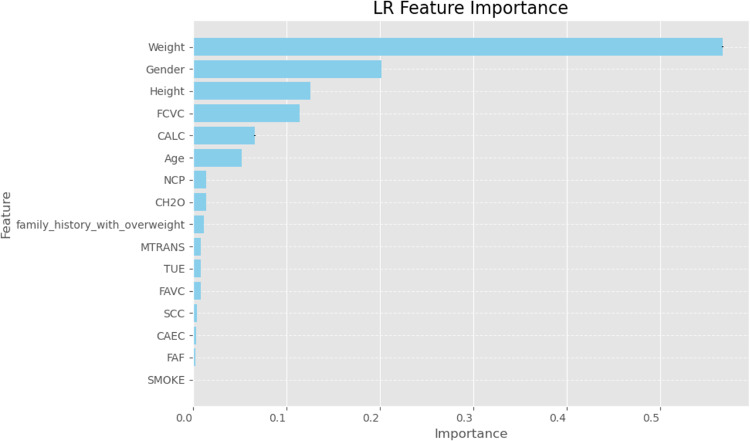
SHAP summary plot of feature importance of LR classifier’s output.

**Fig. 24:**
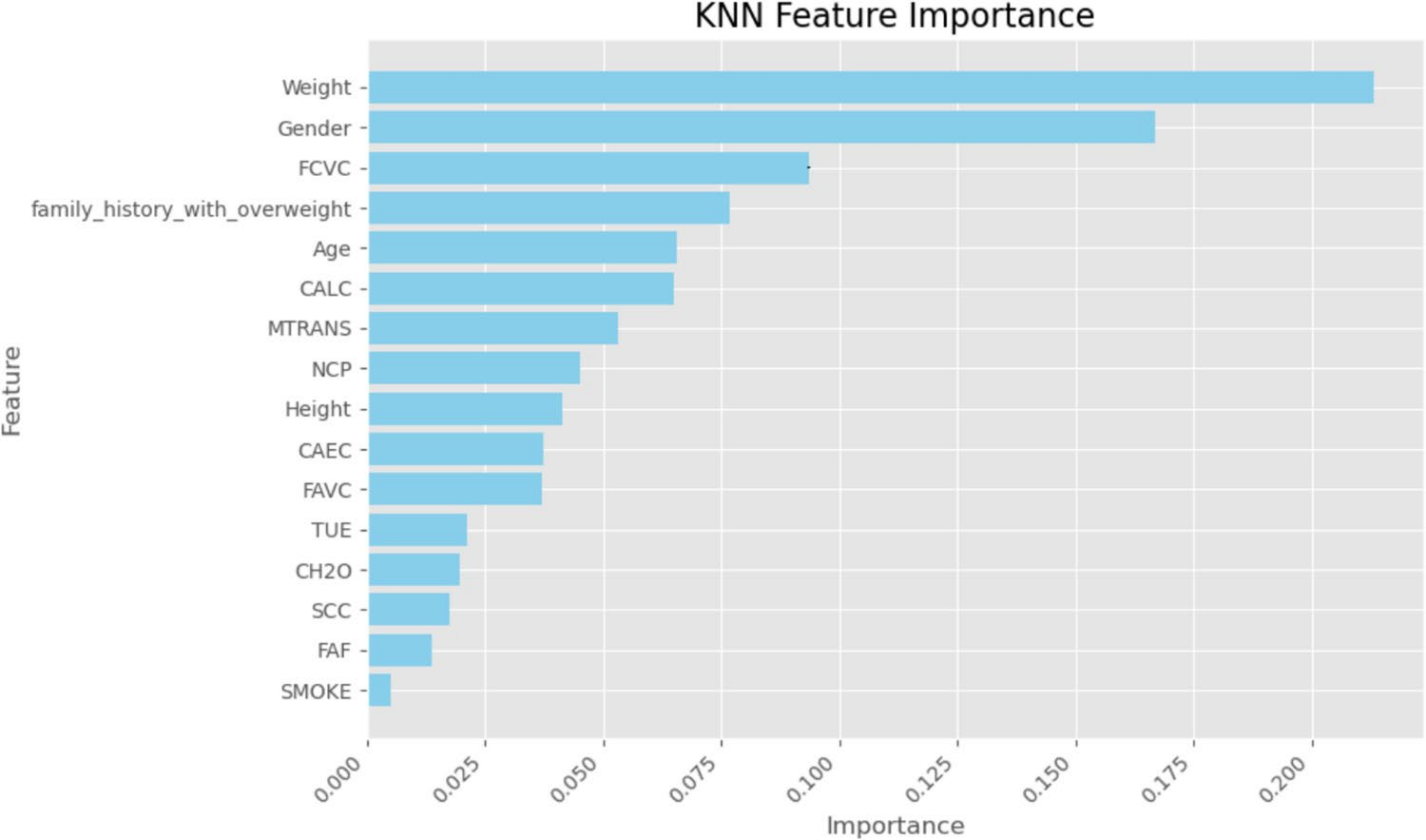
SHAP summary plot of feature importance of KNN classifier’s output.

**Fig. 25:**
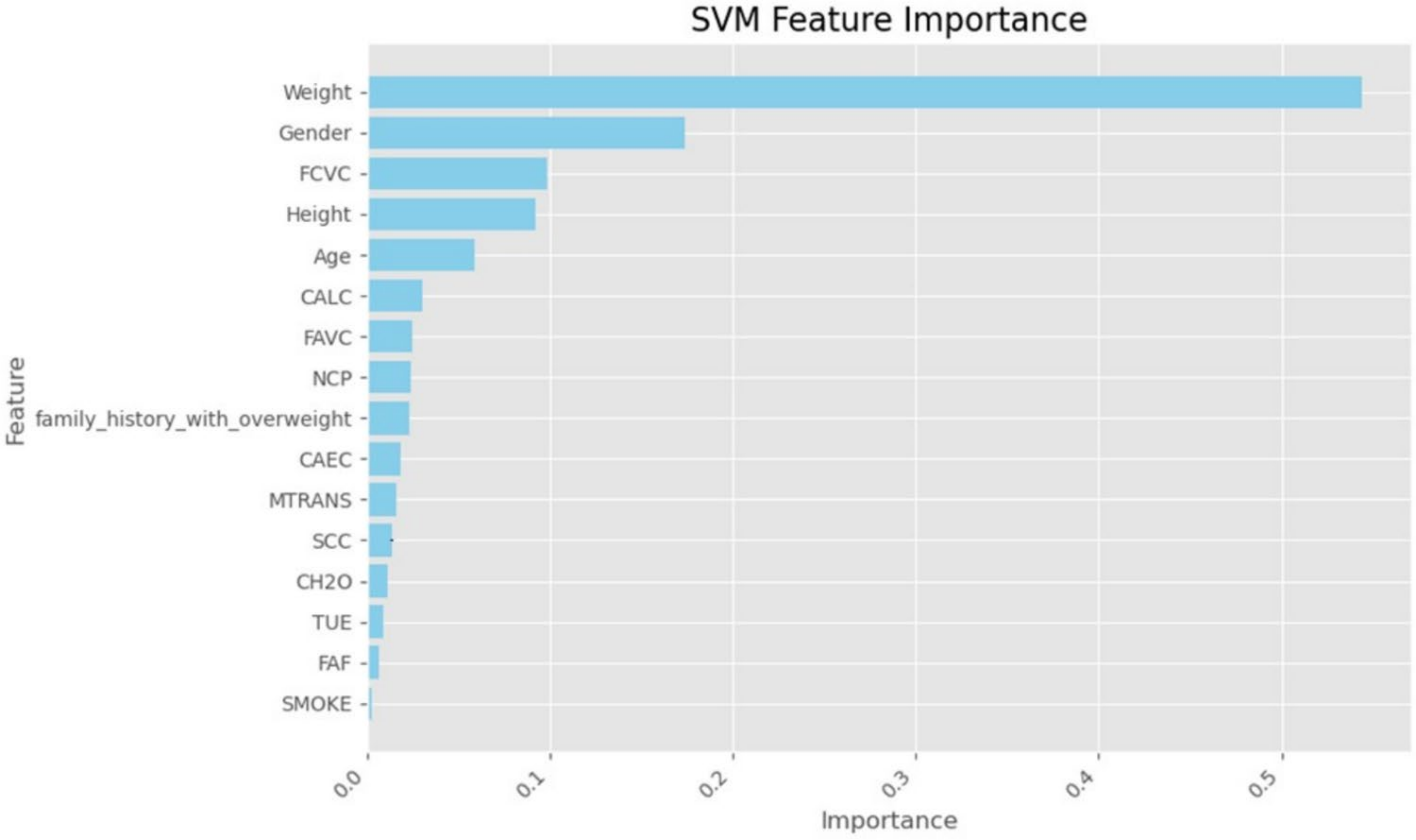
SHAP summary plot of feature importance of SVM classifier’s output.

**Fig. 26:**
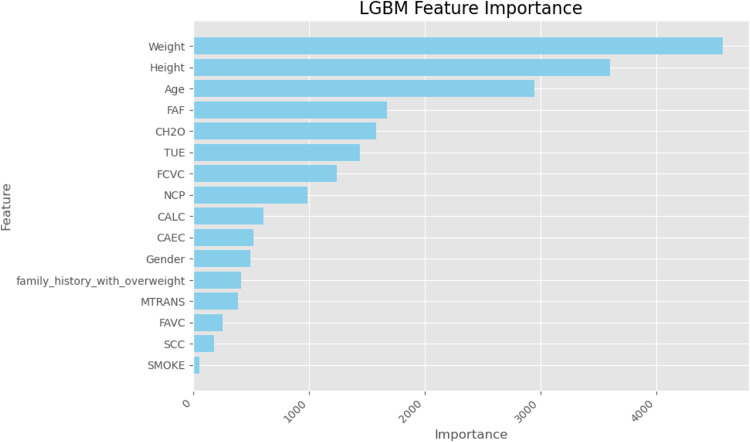
SHAP summary plot of feature importance of LGBM classifier’s output.

**Fig. 27:**
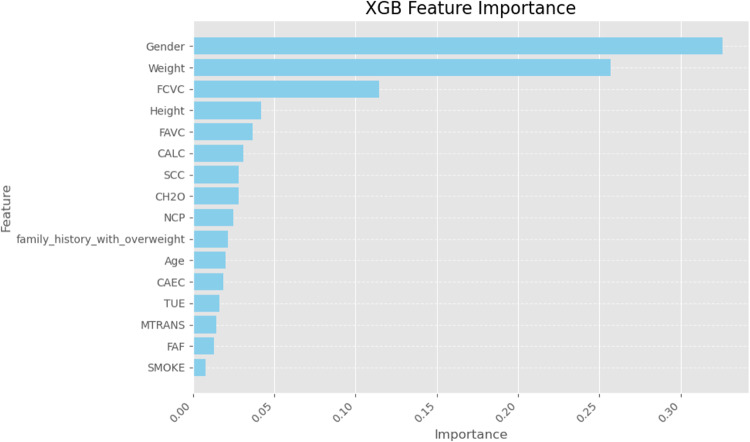
SHAP summary plot of feature importance of XGB classifier’s output.

**Fig. 28:**
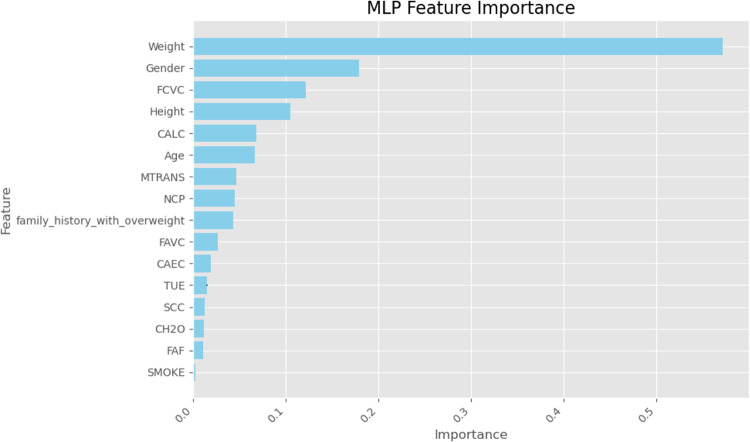
SHAP summary plot of feature importance of MLP classifier’s output.

**Fig. 29:**
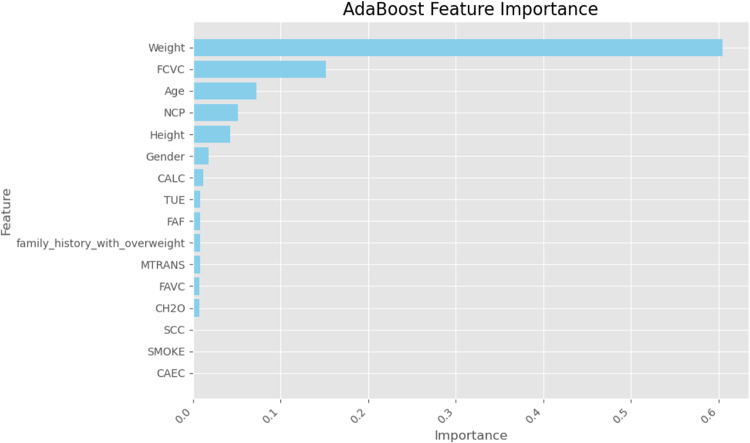
SHAP summary plot of feature importance of AdaBoost classifier’s output.

Finally, we used SHapley Additive exPlanations (SHAP) values to learn more about how the models made their decisions. SHAP analysis sheds light on the relative importance of features for each classifier’s predictions. A SHAP summary plot shown in (Figs. 23, 24, 25, 26, 27, 28, 29) illustrates the average effect of each feature on each model’s predictions. According to (Figs. 23, 24, 25, 26, 27, 28, 29), The main factors impacting the model’s predictions of obesity risk are illuminated by the SHAP value analysis. Interestingly, “weight” turns out to be the most important aspect. The SHAP analysis offers critical insights into each model’s reasoning and underscores the necessity of incorporating both recognized risk factors and potentially innovative indicators such as “time” for a thorough obesity risk evaluation.

### Comparing oberisk with the state of the Art

**Fig. 30:**
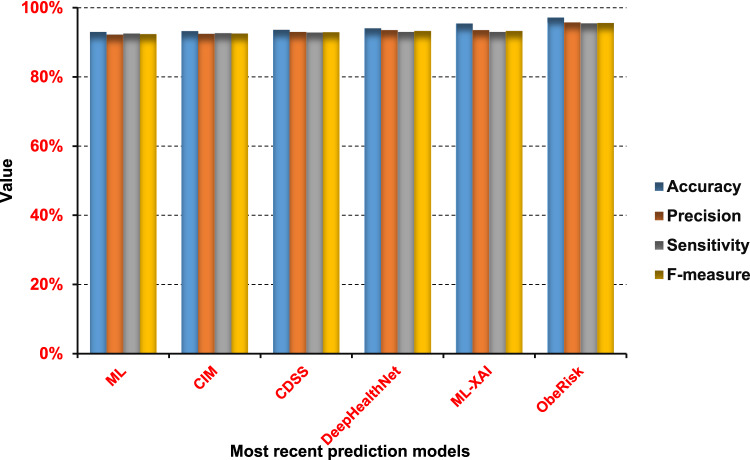
Comparison between the most recent prediction models and ObeRisk.

Via this section, our framework will be evaluated, keeping EC-QBA for feature selection and OPR for prediction, and results were shown in (Table 12). Also, to prove the effectiveness of the proposed ObeRisk, it is compared against the latest methodologies that were discussed in the literature review. These methods are ML^19^, CIM^20^, CDSS^21^, DeepHealthNet^23^, and ML-XAI^26^. All models, including ObeRisk and the comparison models, were evaluated using the same dataset under consistent experimental conditions. Results were shown in (Fig. 30).

**Table 12:** Performance of oberisk in terms of accuracy, precision, sensitivity, and F-measure.

Fold	Accuracy (%)	Precision (%)	Sensitivity (%)	F-measure (%)
1	96.5	95.7	95.5	95.6
2	97.1	96.4	96.02	96.2
3	95.6	93.5	94.01	93.8
4	98.4	97.9	97.3	97.6
5	97.6	96.2	94.9	95.6
6	96.6	94.9	93.71	94.3
7	96.5	93.6	94.4	93.9
8	97.5	96.3	95.8	96.05
9	98.2	96.8	96.1	96.4
10	97.3	96.09	96.9	96.5
Mean ± SD	97.13 ± 0.4	95.7 ± 0.5	95.5 ± 0.4	95.6 ± 0.4

The results for each fold were presented, and the average values were calculated in (Table 12). Our framework’s accuracy, precision, sensitivity, and F-measure for predicting individuals with obesity were displayed in (Table 12). According to Table 12, the performance values of our framework were best for 1, 2, 4, 5, 6, 7, 8, 9, and 10-fold, while they are lowest for 3-fold. The lower precision values were presented in the 3-fold and 7-fold with values of 93.5 and 93.6% in the same order. While the best values were introduced in the 4-fold and 9-fold with values of 97.9 and 96.8% in the same order. Additionally, the lower sensitivity value is introduced in the 6-fold with a value of 93.71%. Additionally, the lower F-measure values were presented in the 3-fold and 7-fold with values of 93.8 and 93.9%, while the best value is introduced in the 4-fold with a value of 97.6%. According to Table 12, our framework is more effective in predicting obese individuals.

As shown in Fig. 30, ObeRisk offers an accuracy of 97.13%, which means it effectively classifies almost all cases. Furthermore, it achieves a precision of 95.7%, indicating that there were few cases of false positives. Additionally, ObeRisk achieves 95.5% sensitivity and 95.6% F-measure, indicating that it is highly effective in identifying actual obesity cases, reducing false negative results, and maintaining a balance between precision and sensitivity. ML-XAI, DeepHealth, CDSS, CIM, and ML introduced accuracies of 95.4, 94, 93.6, 93.2, and 93% in the same order. Additionally, they introduce 93.5, 93.5, 93, 92.4, and 92.2% precision in the same order. Their sensitivity values, in the same order, were 93, 93, 92.8, 92.6, and 92.5%. Its F-measure values were 93.25, 93.25, 92.89, 92.5, and 92.35%, respectively. Finally, ObeRisk demonstrates the highest performance in classifying the obese individuals, outperforming the other recent methodology. ObeRisk relies on an efficient feature selection methodology, specifically EC-QBA.

### Statistical tests

Statistical validation is crucial for evaluating the model’s efficacy. Hence, the Friedman test and the Wilcoxon signed rank test (WSRT) are used to assess and evaluate the predictive potential of the proposed technique^57^. We used WSRT with a 5% significance level and 95% confidence intervals. WSRT results are shown in (Table 13). Assume for the duration of this analysis that the means of the two techniques are not significantly different from one another. Using Minitab, a statistical analysis was carried out. A p-value lower than 0.05 (the 5% significance level) indicates substantial evidence rejecting the null hypothesis, according to the results. This finding suggests that, statistically speaking, the recommended model is different from competing strategies. Therefore, the proposed system outperforms more traditional approaches to obesity prediction.

In addition, the Friedman test metric is used to rank the performance of each model. This metric is nonparametric. This method would determine the difference between the proposed ObeRisk and ML, CIM, CDSS, DeepHealthNet, and ML-XAI at a significant level (α = 0.05). Results were shown in (Table 14). Table 14 shows that the proposed ObeRisk performs better than the most recent models in predicting obesity individuals.

### Comparison with traditional clinical tools

Traditional clinical instruments like BMI are among the most prevalent methods for evaluating obesity risk in routine medical practice. They depend exclusively on height and weight to determine an individual’s health classification. Notwithstanding their user-friendliness and rapid computation, they exhibit considerable deficiencies in accuracy and predictive capability, particularly in scenarios where total body mass is inadequately represented, as seen in athletes or the elderly.

Conversely, the AI-driven ObeRisk model provides a more advanced solution, utilizing a comprehensive array of factors, such as diet, physical activity, age, gender, and overall health condition. It utilizes the EC-QBA feature selection algorithm to identify the most significant factors and subsequently employs a range of ML models, attaining a predictive accuracy of up to 97%, significantly surpassing the accuracy of tools such as the BMI. Table 15 provides a comparison between IBM and our framework.

**Table 13:** WSRT results.

Model 1 vs. Model 2	WSRT	*p*-value	Estimated median difference
ObeRisk vs. ML	0.0	0.00	3.39
ObeRisk vs. CIM	0.0	0.00	3.24
ObeRisk vs. CDSS	0.0	0.00	2.60
ObeRisk vs. DeepHealthNet	0.0	0.00	2.34
ObeRisk vs. ML-XAI	0.0	0.00	2.29

**Table 14:** Friedman mean ranking.

Used model	Rank
ML	6
.CIM	5
CDSS	4
DeepHealthNet	2.6
ML-XAI	2.37
ObeRisk	1

**Table 15:** Comparison between IBM and our framework.

Parameters	ObeRisk	BMI
Used dataset	Lifestyle, age, activity, etc.	Height and Weight only
Interactions between properties	It is modeled using advanced algorithms.	Doesn’t exist
Individual privacy	Customized analysis for each case	Don’t take it into consideration
Scalability	Scalable in digital health systems	Simple, but limited
Transparency and explanation	It can be explained through the selection of features and explanatory models.	clear but limited in meaning
Prediction accuracy	High (97%)	Medium (misclassification can occur)

### Computational efficiency, scalability, and model interpretability

It is crucial to consider the significance of any AI-based predictive model, particularly if it is intended for real-world application. The EC-QBA algorithm incurs higher operational costs compared to alternative feature selection techniques, averaging 13.85 s in execution time and utilizing 136.7 MB of memory, as illustrated in (Table 7). This carefully selected algorithm performs more efficiently. The entropy-based parameter-controlled volume system and mechanical coordination mechanisms render the algorithm more precarious; however, they facilitate information retrieval, thereby enhancing the efficacy of the predictive model. Ultimately, we can enhance the EC-QBA implementation or employ hardware acceleration techniques such as GPU computing for its execution.

Scalability remains crucial, particularly as healthcare datasets continue to expand in size and complexity. EC-QBA employs a population-based architecture that inherently facilitates parallel processing. This indicates that the algorithm can manage increased data volumes without necessitating additional simultaneous calculations. The modular architecture of the ObeRisk model facilitates its application in distributed or cloud computing environments, essential for handling the extensive datasets prevalent in clinical and epidemiological research. However, experimental evaluations on larger and more diverse datasets are essential to confirm these scalability claims and identify possible bottlenecks.

Additionally, interpretability is essential for cultivating trust and the implementation of models in clinical settings, as understanding the reasoning behind predictions impacts medical decision-making. The ObeRisk model employs ensemble learning techniques, which may obscure clarity; however, we enhanced interpretability by analyzing the significance of features derived from specific models and utilizing EC-QBA’s capacity to identify the most critical features. This dual approach assists physicians in comprehending the primary factors that increase the likelihood of obesity, thereby facilitating the understanding of intricate models. Interpretable AI tools, such as SHAP, enhance clarity by providing both local and global interpretations of model predictions.

### Limitations and potential biases

A crucial element in predictive modeling is the potential biases inherent in the dataset, which may negatively impact the model’s equity and generalizability. This study uncovered a gender disparity in the dataset employed for predicting obesity risk, potentially biasing predictions towards the most representative group. This disproportionate representation may impair the model’s efficacy for the underrepresented gender, resulting in diminished accuracy and reliability of predictions for those groups.

To mitigate this risk, the EC-QBA algorithm selects features with substantial predictive power throughout the entire dataset. This mitigates bias induced by features that are either irrelevant or excessively noisy. In Future, additional methodologies, such as resampling techniques or fairness-aware algorithms, will be used to ensure equitable and balanced model performance across various demographic groups. Additionally, examining the model outputs for each gender category individually can facilitate the identification of disparities and propose enhancements. To utilize reliable AI systems in healthcare, it is essential to identify and rectify biases in data. Accurate predictions significantly influence patient outcomes.

## Conclusions and future works

In this paper, we proposed a machine learning framework for predicting obesity susceptibility called ObeRisk. In fact, ObeRisk composed of three main stages: (i) Preprocessing Stage (PS) where the used dataset, containing information about individuals like weight, height, lifestyle, etc., was preprocessed by removing or filling missing values, removing outliers, encoding categorical features, and the normalization process. (ii) Feature Stage (FS) where the most important features were selected using the proposed Entropy-Controlled Quantum Bat Algorithm (EC-QBA). EC-QBA adjusted BA parameters using Shannon entropy and updated the next position of BA in local search using a quantum mechanism. (iii) Obesity Risk Prediction (OPR) where the selected features were fed into the several ML algorithms, with the final decision based on majority voting. Experimental results demonstrated the effectiveness of our framework when comparing it with the recent prediction methods.

Consequently, ObeRisk model represents an important step toward using predictive analytics to help address the growing public health challenges associated with obesity. The model effectively identifies individuals at greatest risk for obesity-related complications by integrating diverse clinical, behavioral, and demographic data, facilitating early intervention and more appropriate healthcare planning. Despite the encouraging outcomes of our framework, several limitations need attention:

- Feature availability: the framework assumes the availability of certain personal and lifestyle data, such as weight, height, and activity level. In reality, this information may be absent, inaccurate, or outdated.
- Computational complexity: EC-QBA is effective; however, it introduces additional computational complexity owing to entropy calculations and quantum-based updates. Additionally, using several ML models can demand significant computational resources.

As future work, various combinations of ML models could be explored to identify the minimum set that achieves comparable voting accuracy. This approach would improve computational efficiency while maintaining prediction accuracy. Additionally, we aim to test our framework on other obesity-related and non-obesity datasets. Furthermore, we aim to evaluate our framework on an external dataset, such as NHANES, which would be beneficial for assessing its generalizability across diverse populations. Finally, we plan to enhance the ObeRisk model by incorporating sub-analyses based on gender, age group, and potentially ethnic background, aiming to augment the model’s transparency and equity.

## Supplementary Information

Below is the link to the electronic supplementary material.

Supplementary Material 1

## Data Availability

The used dataset is available at https://www.kaggle.com/datasets/jpkochar/obesity-risk-dataset.
