# Supplementary material for: Machine learning framework for predicting susceptibility to obesity
Source: Sci Rep. 2025 Oct 8;15:35040. doi: 10.1038/s41598-025-20505-9 (PMC12508184; doi:10.1038/s41598-025-20505-9)
Supplement: Supplementary file 1 — Supplementary Material 1 [file 41598_2025_20505_MOESM1_ESM.docx]

**Supplementary Materials – Obesity Statistics and WHO Classification Criteria**

According to the World Health Organization (WHO), 65 percent of adults, 34 percent of teenagers, and 39 percent of children are overweight or obese [1]. Worldwide, more and more people are becoming overweight or obese. In fact, according to WHO predictions, around 167 million people (including children and adults) will experience health problems related to obesity by the year 2025. According to research, the number of people who are overweight is projected to surpass 2.16 billion by 2030, with 1.12 billion being classified as obese. Therefore, epidemiological studies have shown that being overweight or obese is associated with an increased risk of a number of health problems [2,3].

The primary measure for determining if a person's weight is normal, overweight, or obese is the Body Mass Index (BMI). A person's BMI is calculated by dividing their height in meters squared by their weight in kilograms. In adults, a BMI under 18 kg/m² is classified as underweight. A BMI between 18 and 25 kg/m² is considered normal weight. A BMI above 25 kg/m², up to 30 kg/m², is categorized as overweight and obese, respectively. The increasing rates of obesity pose a major threat to public health in many countries. This trend results in higher mortality rates, a lower quality of life for individuals, and increased costs for society [2].

Classifications of BMI used to evaluate obesity in newborns, children, and teenagers differ according to gender and age [1,4].

- The WHO uses the following criteria to describe overweight and obesity in adults: BMI values of 25 or above indicate overweight status, while BMI values of 30 or above indicate obesity. In order to define childhood obesity and overweight, age is a key factor.
- People who are less than 5 years old: If a child's weight is more than two standard deviations higher than the median of the WHO's Child Growth Standards, they are classified as overweight. Obesity is defined as a weight-for-height ratio higher than the median by more than three standard deviations.
- People whose ages range from 5 to 19: The following criteria are used to define overweight and obesity in this age bracket: Any BMI for a given age that is more than one standard deviation higher than the median of the WHO's Growth Reference indicates overweight. The WHO Growth Reference median BMI plus two standard deviations is considered obesity.

The health risks linked to being overweight and obese are becoming more clear and understood [1,5].

- About 3.7 million deaths in 2021 were attributed to noncommunicable diseases (NCDs), such as cardiovascular disease, diabetes, cancer, neurological disorders, chronic obstructive pulmonary disease, and gastrointestinal disorders, which are all exacerbated by a higher body mass index (1).
- Multiple non-communicable diseases, including type 2 diabetes and cardiovascular disease, are more likely to occur at an earlier age in children and adolescents who are overweight, which has immediate negative effects on their health. Stigma, prejudice, and bullying amplify the negative psychosocial effects of childhood and adolescent obesity, which impact academic performance and general quality of life. Childhood obesity greatly increases the risk of adult obesity and the prevalence of non-communicable diseases.
- There will be major monetary repercussions from the obesity epidemic. Worldwide, the monetary toll of overweight and obesity is expected to reach $3 trillion annually by 2030 and exceed $18 trillion by 2060 if current trends continue.

The increasing prevalence of obesity in low- and middle-income nations, particularly among lower socio-economic demographics, is rapidly transforming a concern that is previously linked solely to high-income countries.

Overweight, obesity, and their associated NCDs are predominantly preventable and manageable. Individuals can reduce their risk by applying preventive measures from pre-conception through early childhood [1,5]. These encompass:

- Support the optimal weight gain of the woman during pregnancy; additionally, follow the exclusive breastfeeding during the first 6 months after birth and continue to breastfeed until 24 months or even longer;
- Drive the children to become healthy eaters, physically active, cut down on sedentary behavior, and sleep properly; however, be conscious of their weight today.
- Limit screen time; cut back on sugary drinks and calorie-heavy foods; encourage healthier eating habits; promote a healthy lifestyle (including regular exercise, a well-rounded diet, enough sleep, not smoking or drinking, and learning to control one's emotions); reduce energy intake from fats and sugars; increase consumption of plant-based foods, legumes, whole grains, and nuts; and engage in regular physical activity.
